# Review of Biomarkers in Ocular Matrices: Challenges and Opportunities

**DOI:** 10.1007/s11095-019-2569-8

**Published:** 2019-01-23

**Authors:** Mitalee Tamhane, Sara Cabrera-Ghayouri, Grigor Abelian, Veena Viswanath

**Affiliations:** 1 Clinical Pharmacology, Allergan plc, 2525 Dupont Drive, Irvine, California 92612 USA; 2 Biological Research, Allergan plc, 2525 Dupont Drive, Irvine, California 92612 USA; 3 Clinical Pharmacology and Pharmacometrics, Bristol-Myers Squibb, Lawrence Township, New Jersey 08648 USA

**Keywords:** aqueous humor, biomarkers, conjunctiva, ocular diseases, tears, vitreous

## Abstract

Biomarkers provide a powerful and dynamic approach to improve our understanding of the mechanisms underlying ocular diseases with applications in diagnosis, disease modulation or for predicting and monitoring of clinical response to treatment. Defined as measurable indicator of normal or pathological processes, biomarker evaluation has been used extensively in drug development within clinical settings to better comprehend effectiveness of treatment in ocular diseases. Biomarkers in the eye have the advantage of access to multiple ocular matrices via minimally invasive methods. Repeat sampling for biomarker assessment has enabled reproducible objective measures of disease process or biological responses to a drug treatment. This review describes the usage of biomarkers with respect to four commonly sampled ocular matrices in clinic: tears, conjunctiva, aqueous humor and vitreous. Issues that affect the evaluation of biomarkers are discussed along with opportunities to leverage biomarkers such that ultimately, they can be used for customized targeted therapy.

## Introduction

The eye is a complex sensory organ, capable of receiving light and converting it into electrical impulses which are transmitted to the brain via the optic nerve, resulting in visual perception. Broadly, it can be divided into the anterior and the posterior segments. The anterior segment is comprised of the cornea, conjunctiva, aqueous humor, iris, ciliary body and lens, while the posterior segment is comprised of the sclera, choroid, retina and vitreous. The ocular surface (cornea, conjunctiva and meibomian glands), the lacrimal glands and the interconnecting sensory and motor nerves constitute an integrated functional unit known as ‘lacrimal functional unit’ (LFU) (1). This functional unit controls the volume and composition of the tear film which keeps the ocular surface hydrated and is responsible for maintenance of ocular health and homeostasis. The intraocular pressure which is the tension exerted by the contents of the globe on the corneoscleral envelope, maintains the shape of the eye, and is essential for normal optics of the eye (2). While the vitreous acts as an optical media, the retina is critical in terms of converting light to neuronal impulses that traverse the visual pathway to reach the brain. Furthermore, several factors make the eye resilient to disease or injury. Outer structural components such as sclera and cornea minimize internal injury. Blood-aqueous and blood-retinal barriers promote immune privilege and ocular homeostasis. Several intraocular immune modulators and cells manage inflammation in an effort to reduce potential tissue damage (3). Various ocular disorders resulting in an impairment in these critical functions or damage to any of the ocular matrices could ultimately cause loss of vision. Dry eye disease (DED), bacterial or viral infections, inflammatory conditions such as blepharitis, atopic keratoconjunctivitis (AKC) and vernal keratoconjunctivitis (VKC) are some of the common disorders affecting the anterior or ocular surface tissues. Similarly, common diseases impacting the posterior ocular segment are glaucoma, macular edema, diabetic macular edema (DME), proliferative vitreoretinopathy, age-related macular degeneration (AMD), endophthalmitis and diabetic vitreoretinopathies, could result in vision loss if left untreated (4). To develop an effective treatment for diseases impacting both anterior and posterior tissues, it is critical not only to understand the disease pathophysiology and progression, but also be able to measure modulation in disease due to pharmacological intervention. For these reasons and to ensure a successful drug development process, development of a biomarker as a specific and sensitive tool becomes critical.

The biomarker definitions working group, convened by National Institutes of Health (NIH), defined a biomarker as “*a characteristic that is objectively measured and evaluated as an indicator of normal biological processes, pathogenic processes, or pharmacologic responses to a therapeutic intervention”* (5). More recently, in the spring of 2015 the FDA-NIH Joint Leadership Council developed the BEST (Biomarkers, Endpoints, and other Tools) Resource, which slightly modified the original biomarker definition to “*a defined characteristic that is measured as an indicator of normal biological processes, pathogenic processes, or responses to an exposure or intervention, including therapeutic interventions*” (6). This resource also outlined the different types of biomarkers – molecular, histologic, radiographic and physiologic and classified them into 7 main categories: diagnostic, prognostic, monitoring, predictive, response, safety and susceptibility/risk. Biomarkers have been used for patient selection to enrich a clinical study, for classification or staging of a disease, as an indicator of disease modulation or for predicting and monitoring of clinical response to an intervention. Most of the times, biomarkers are exploratory in nature and their development has been initiated in preclinical models and progressed into evaluation in clinic.

For ocular diseases, an effective biomarker should be easy to measure and collected from target tissue of interest rather than from blood or urine. In animal models of ocular disorders, a variety of ocular matrices can be harvested and analyzed for biomarkers, but for implementation of that biomarker measurement in clinic, the type of ocular matrix to be sampled is a key consideration. In humans, ocular matrices that are most readily accessible are tears and ocular surface tissues such as cornea and conjunctiva. These ocular matrices provide valuable information regarding anterior segment disorders, but it is the aqueous humor (AH) and vitreous which are more suitable matrices for evaluation of relevant biomarkers for posterior segment disorders. These are difficult to access, more invasive and require small procedure in clinic to facilitate sampling. This review focusses on the established and novel biomarkers in clinical studies, evaluated in the ocular matrices which have been most commonly sampled: tears, conjunctival cells, AH and vitreous. Biomarker evaluation in these matrices has provided valuable insight into diagnosis of disease, progression or modulation of disease with and without pharmaceutical intervention, thus making ocular biomarker assessment critical component of ophthalmic drug discovery and development. For the purposes of this review, only those biomarkers relevant in ocular diseases, have been discussed. In addition, there is discussion regarding the collection techniques and analytical procedures along with associated challenges and opportunities.

## Tears

The Tear Film and Ocular Surface Society (TFOS) in a report entitled as: “Dry Eye Workshop II (DEWS II)” defined a stable tear film requisite for a healthy ocular surface as a complex fluid composed of three key elements: 1) A mucin layer composed of high molecular weight (Mw) glycoproteins that cover the ocular surface and lower the hydrophobicity of the epithelial cells; 2) A lubricating aqueous layer that provides some nutrients, antimicrobial proteins and suitable osmolarity; 3) A lipid layer that prevents loss of the aqueous layer (7). It is now believed that the mucin and the aqueous layers are a single layer that form the muco-aqueous layer. In addition to lubrication of the eyelids during blinking, the primary function of this complex fluid is to maintain the health and homeostasis of the ocular surface including the cornea and the conjunctiva and to preserve the high optical quality of the corneal surface. It has now been demonstrated that tears contain thousands of molecules that include lipids, electrolytes, proteins, peptides and multiple small molecule metabolites secreted from multiple sources such as the lacrimal glands, Meibomian glands, goblet cells and ocular surface epithelial and nerve cells (8).

Tears are classified into four broad types based on the mode of production: 1) basal tears which are the tears that cover our eyes continually and are critical for ocular surface health. These tears are deficient in DED and other autoimmune diseases like rheumatoid arthritis, Sjögren’s Syndrome (SS), lupus etc; 2) reflex tears are produced upon stimulation of the reflex arc such as nasal stimulation of the sneeze reflex; 3) closed eye tears are tears produced during sleep that is now believed to be critical for clearing debris, maintain homeostasis and can be collected immediately after a period of sleep from the ocular surface and 4) emotional tears are induced tears due to emotions such as sadness or happiness (7).

Despite the small volume available for sampling, the tear fluid is a key source of biological material that is used to evaluate health and pathology of the eye, using minimally invasive techniques. Tear fluid has the advantage of being proximal to the disease site on the ocular surface which makes it ideal to evaluate the composition to identify reliable biomarkers of ocular surface diseases such as DED, VKC, AKC, SS, Meibomian gland dysfunction (MGD), ocular graft *versus* host disease (OGVHD) in addition to retinal diseases, thyroid-associated ophthalmopathy (TAO) and extraocular diseases. Progress in the search for tear biomarkers in various diseases has been reviewed before (8–14). Multiple methods have been employed to identify reliable biomarkers in tears and are reviewed below. Table I summarizes the key biomarkers in tears.

**Table I:** Summary of Key Biomarkers in Human Tears

Biomarker	Ocular Disease	Function of the biomarker^a^	References
Lactoferrin	DED	Multifunctional iron-binding glycoprotein; normal cell growth and is critical for maintaining normal ocular surface health	(15,17,23,24,79,81,82,86)
MMP-9	DED, SS, OGVHD	Endopeptidase; key role in extracellular matrix remodeling of the injured corneal surface	(31–38,60,61,63,65,75,76,118,147,152)
Inflammatory cytokines			
IFN-γ	DED, SS	Immunoregulatory cytokine shown to play a role in TH 1-driven immune response at the ocular surface	(31,42,45–48,50,53,54,57,58,60,62,63,72,73,118)
TNF-α	DED	Cytokine secreted by macrophages involved in inducing cell death of certain tumor lines; may stimulate cell proliferation/induce cell differentiation under different conditions	(42,47–49,51,53–55,57,58,60,61,63,72,73)
IL-1α, IL-1β	DED	IL-1α: Proinflammatory cytokine involved in hematopoiesis IL-1β:Proinflammatory cytokine that promotes Th17 differentiation	(42,47,48,58,60,61,63–65,72,118)
*Th-17 associated Cytokines* (IL-6, IL-17A, IL-17F, and IL-22)	DED, SS	Th17 cells are a subset of CD4+ T helper cells; critical in maintaining the chronic and relapsing phase of multiple immune diseases IL-17: Immunoregulatory cytokine involved in inducing production of proinflammatory and hematopoietic cytokines IL-22: Proinflammatory cytokine secreted by NK and T cells IL-6: Inducer of the acute phase response and involved in the final differentiation of B cells	(31,42,46–49,51,58,61–64,66–73,75,76,91,118)
Chemokines			
IL-8/ CXCL8	DED	Directs the migration of neutrophils, basophils and T lymphocytes mediating innate immune and angiogenic response	(42,46–49,56,58,61,63,72,73)
MIP-1α/ CCL3, MIP-1β/CCL4, RANTES/CCL5, Fractalkine/CX3CL1, CXCL9, CXCL10, CXCL11, MCP-1/CCL2	SS, DED	MIP-1α/ CCL3: Proinflammatory chemokine expressed on T cells MIP-1β/CCL4: Chemoattractant for NK cells and monocytes; proinflammatory RANTES/CCL5: Chemoattractant for monocytes, memory T-helper cells and eosinophils; induces release of histamine Fractalkine/CX3CL1: Chemokine involved in neutrophil chemotaxis CXCL9: Inflammatory mediator; chemotactic for activated T cells CXCL10: Chemotactic for monocytes and T-lymphocytes CXCL11: Induces calcium release in activated T-cells; acts as a chemotactic for interleukin activated T-cells MCP-1/CCL2: Attractant for monocytes and basophils	(43,46–48,52,58,61,63,72,76)
Protein biomarkers			
EGF	SS, Stevens-Johnson syndrome, DED	Stimulant for the growth of various epidermal and epithelial tissues	(46,49,58,63,74–76,79)
LPRR4, LPRR3, nasopharyngeal carcinoma associated PRP4 and α-1 antitrypsin	DED, SS, MGD	LPRR4: Plays a role in protective functions in the eye LPRR3: Plays a role in protective functions of the eye PRP4: Inhibitors of calcium phosphate precipitation; binds to bacterial pathogens and minerals/tannins α-1 antitrypsin: Inhibits serine proteases, trypsin, chymotrypsin, and plasminogen activators. Has proteolytic activity against insulin and plasmin	(15,21,22,80–82,85,86)
PIP (prolactin- inducible protein)	DED	Regulates water transport in apocrine glands, binds to IgG CD4 T-cell receptors	(23,86)
LCN-1 (Lipocalin-1)	DED	Major tear protein; principle lipid binding protein on epithelial cell surfaces	(15,22,23,42,45,77,81,82,86)
α-enolase	DED	Multifunctional enzyme that plays a role in allergic responses through stimulation of Ig production	(15,23,85)
S100 family of proteins: S100A8/Calgranulin A, S100A9/Calgranulin B, S100A4 and S100A11	DED	S100A8: Calcium binding protein; regulates inflammatory processes and immune responses S100A9: Calcium binding protein; regulates inflammatory processes and immune responses S100A4: Calcium binding protein; involved in cell growth and motility S100A11: Calcium binding protein; facilitates differentiation/cornification of keratinocytes	(15,21,23,45,78,82,85)
Annexin A1 (ANXA1), Annexin A11 (ANXA11),	DED	ANXA1: Effector of glucocorticoid-mediated responses and regulator of inflammatory processes ANXA11: Calcium-dependent phospholipid binding protein	(78,82,85)
MUC5AC	DED, SS	Gel-forming glycoprotein; protects mucosa from infection and chemical damage	(88–92)
Cathepsin S	SS	lysosomal cysteine endopeptidase	(87,88,93)
Neuromediators: substance P, NGF, VIP, CGRP	DED	Substance P: neuropeptide; vasodilator; key first responder to extreme stressors NGF: Activator of cellular signaling cascades through tyrosine kinase receptors VIP: Used by neurons to communicate with postsynaptic targets to regulate circadian rhythm CGRP: Causes cerebral vasodilation; may have a neurotransmitter/neuromodulator role	(95,96)
Other potential biomarkers: IgE, Tryptase, Histamine, ECP	Ocular allergies	IgE: Antibody commonly seen in response to allergens Tryptase: Protease present in mast cells and secreted in response to a coupled activation-degranulation response Histamine: Compound released by cells in response to injury and inflammatory reactions; causes contractions of smooth muscle and dilation of capillaries ECP: Released during degranulation of eosinophils; related to inflammation and asthma	(25,45,47,50)

^a^function has been summarized based on information in references, and from website search: uniprot.org and google.com

### Collection of Tears and Analytical Methodology

Tears are collected non-invasively through multiple methods using Schirmer strips, other absorbent materials such as minisponges, fire-polished microcapillary tubes and eye wash. It has been shown that the tear collection methodology differences, and storage conditions can contribute to the differences observed between different studies (15). Proteomic studies have primarily used microcapillary methods for tear collection, although a few studies have used Schirmer strips. The critical factor to keep in mind during tear collection is to not activate the corneal nerves and induce reflex tearing which can alter the composition of the tear fluid (16,17). In addition, external factors like use of topical anesthesia, contact lens wear, use of artificial tears, collection from open *versus* closed eyes etc. significantly impact tear composition. Several methodologies such as evaluation of tear proteome, lipidome, metabolome, and multiplex analysis of inflammatory mediators are utilized to evaluate the tear composition.

Multiplex assay technologies such as cytometric bead array (CBA) -Luminex, DropArray have made possible analysis of multiple molecules in small sample volume of tears (18–20). Advances in proteomic, lipidomic and metabolomic analyses in tears have been made possible through improvements in Mass spectrometry (MS) and bioinformatic analysis methods of large datasets. Different mass spectrometric techniques have been used to analyze tears including surface-enhanced laser desorption ionization-time of flight (SELDI-TOF-MS) and matrix assisted laser desorption ionization-time of flight (MALDI-TOF-MS) (21,22). Recently isobaric tags for relative and absolute quantitation (iTRAQ) technology coupled to 2D-nanoLC-MS/MS has improved quantitative accuracy, coverage and robustness in evaluation of tear proteomics (23).

### Biomarkers in Tears

#### Point of Care Biomarkers in Tears

There are a few FDA approved point of care biomarkers used in the clinical setting for the diagnosis and treatment of DED. One of the first devices to get approved was the Advanced Tear Diagnostics’ ocular lactoferrin tear test. Lactoferrin is a multifunctional iron-binding glycoprotein, and low levels of lactoferrin are believed to indicate aqueous deficient DED (24). It is well established that lactoferrin plays an important role in modulation of ocular inflammatory response and normal cell growth and is critical for maintaining normal ocular surface health. It is one of the most abundant proteins in the tears and lower levels have been reported in herpes simplex keratitis, systemic infections in addition to DED. An additional point of care test was the Total Immunoglobulin E (IgE) diagnostic kit which is a quantitative diagnostic kit utilized to confirm the diagnosis of allergic conjunctivitis (25). Two tests, namely, the measurement of tear osmolarity and the measurement of tear levels of matrix metalloproteinase-9 (MMP-9) are currently widely used in clinical settings for DED diagnosis and are discussed below.

#### Tear Osmolarity

Change in tear osmolarity has been widely used as an important tool in the diagnosis of DED and the Tearlab osmolarity test is a device used in clinical practice as a semi-automatic method for measuring tear osmolarity (26). Changes in concentration of electrolytes and proteins in the muco-aqueous layer, an insufficient or unstable tear film, increased tear evaporation rates are all postulated to contribute to hyperosmolarity of the tear film. A range of osmolarity of 308 mOsm/L to >316 mOsm/L is used as a cutoff for diagnosing DED (27–30). Given the variability, it has been observed that tear hyperosmolarity is not evident in all dry eye patients. However, if it can be detected, it is indicative of significant pathology.

#### Matrix Metalloproteinase-9

Inflammatory mechanisms are the key drivers of ocular surface diseases such as DED, SS, and OGVHD. MMP-9 is an endopeptidase which plays a key role in extracellular matrix remodeling of the injured corneal surface. Multiple studies have demonstrated that levels of MMP-9 in tears are higher in DED, SS and OGVHD patients (31–33). Based on these results a point of care test for MMP-9 called InflammaDry was FDA-approved, and is subsequently used in clinical practice to evaluate inflammatory status of the eye to enable decision to treat with an anti-inflammatory therapy (34–37). This diagnostic tool is believed to be suited for the detection of moderate to severe dry eye patients, however it is challenging to use this test in subjects with no previous dry eye diagnosis or those who have mild disease (37,38).

#### Other Biomarkers Proposed in Tears

#### Inflammatory Mediators

Inflammation and immune-mediated mechanisms are central mechanisms that contribute to etiology of DED, SS, ocular allergy, OGVHD and other inflammatory ocular surface diseases. Cytokines and chemokines are endogenous inflammatory mediators secreted by a wide variety of cells and their presence in normal tears have been described (39–41). Multiple studies have demonstrated that various inflammatory and immune-related cytokines/chemokines are significantly increased in tears in DED, SS, ocular allergy, OGVHD and other inflammatory conditions (7,31,42–55). In addition to the cytokines and chemokines, additional biomarkers have been proposed in ocular allergy to evaluate extent of neutrophil, eosinophil and lymphocyte infiltration by measuring tear fluid levels of IgE, tryptase, histamine and eosinophilic cationic protein (ECP) (25). The tear levels of several of these inflammatory molecules have been correlated to clinical parameters and/or disease severity further adding to the value of these molecules as potential biomarkers to evaluate diseases of ocular surface inflammation. It has also been reported that several of these tear cytokine and chemokine levels do not show significant inter-day variation and show good intra-subject repeatability in healthy subjects (39,56). . However, a wide range of concentrations have been observed for these cytokines and chemokines between the different studies, which has been reviewed in Roy *et al.* (14). For example, Interleukin-8 (IL-8) which has been reported to be elevated in tears of dry eye patients range in concentrations between 74+55pg/ml to 6518+4510 pg/ml when compared to 176+72pg/ml to 1150+50pg/ml in normal tears (57–61). These variations in concentrations are attributed to differences in collection, sample processing and analysis methods, clinical criteria, stringency in data analysis etc. This has made comparison of the absolute concentrations of these cytokines and chemokines between different studies challenging. Development of a validated point of care diagnostic tool with the key inflammatory mediators would add significant value in clinical research and therapeutic treatment strategies for ocular surface inflammatory diseases. In the following 2 subsections, we discuss a few cytokine and chemokine changes that have been reported particularly in DED and SS.

#### Inflammatory Cytokines

Interferon-gamma (IFN-γ) is the signature cytokine that is secreted from T-helper 1 (Th-1) cells and is also produced by other cells such as Natural Killer (NK) cells, epithelial cells etc. and is associated with variety of immune functions such as recruitment and polarization of Cluster of Differentiation 4 positive (CD4+) Th1 cells and induction of multiple Th1 cytokines and chemokines. Elevated levels of IFN-γ in tears of patients from DED and SS has been reported (42,62,63). Many of the same studies and other groups have also shown elevated levels of tumor necrosis factor-alpha (TNF-α) which is thought to represent a measure of the general inflammatory status of the ocular surface in sub-sets of DED (48,51,58). TNF-α has also been shown to be significantly higher in tears from TAO patients when compared to controls (54,55). The proinflammatory cytokine Interleukin-1 (IL-1) which includes two forms- Interleukin-1alpha (IL-1α) and Interleukin-1beta (IL-1β) has been detected in human tear fluid (39,64). Clinical studies have reported that tears of dry eye patients show increased levels of IL-1α and mature IL-1β which correlated to corneal fluorescein staining (42,48,65). Similar increased levels of inflammatory cytokines have also been reported in active TAO patients indicating that measurement of tear cytokine levels might be a useful diagnostic tool in multiple ocular inflammatory conditions (54,55).

T-helper 17 (Th-17) cell associated cytokines, namely Interleukin-6 (IL-6), Interleukin-17A (IL-17A), Interleukin-17F (IL-17F) and Interleukin-22 (IL-22) are a subset of CD4+ T helper cells which have been shown to play an important role in maintaining the chronic and relapsing phase of multiple immune diseases including DED and SS (66–68). IL-17 and IL-22 are the effector cytokines of the Th-17 cells and have been reported to be elevated in dry eye patients, with or without Sjögren’s, when compared to normal subjects (69). Furthermore, these two cytokines are highest in tears of Sjögren’s patients indicating that Th-17 cytokines play a role in ocular surface inflammation and pathogenesis of disease(62,70). Another key cytokine that has been evaluated in multiple studies is Interleukin-6 (IL-6) which has both pro-and anti-inflammatory roles and may represent a biomarker for evaluation of treatment effects as levels of IL-6 have been reported to decrease after treatment with 0.05% Cyclosporine (71). Yoon *et al*, reported an increase in levels of IL-6 in tears of dry eye patients and that it is associated with severity of disease correlating with Tear film break-up time (TBUT), Schirmer test, goblet cell density and other measures (51).

#### Chemokines

Interleukin-8 (IL-8), also called as chemokine (C-X-C motif) ligand 8 (CXCL8), a key cytokine that directs the migration of neutrophils, basophils and T-lymphocytes by mediating innate immune and angiogenic response, has consistently been reported to be elevated in tears of dry eye patients (42,58,63,72,73). Pinto-Fraga *et al*. reported in a study, that IL-8 long with other inflammatory mediators such as epidermal growth factor (EGF), IFN-γ, Interleukin-2 (IL-2), regulated on activation, normal T cell expressed and secreted/ chemokine (C-C motif) ligand 5 (RANTES/CCL5) and MMP-9 could represent biomarkers of disease severity in DED (63). Additionally, multiple studies have shown alterations in tear EGF levels and in MGD it has been associated with corneal subepithelial fibrosis and other changes at the lid margins (46,49,58,63,74–76). It has also been reported that exposure of dry eye patients to controlled desiccating conditions does not alter the levels of tear IL-8 (76). Several studies in dry eye patients have demonstrated elevated levels of tear chemokines , such as macrophage inflammatory protein 1 alpha/chemokine (C-C motif) ligand 3 (MIP-1α/ CCL3), macrophage inflammatory protein 1 beta/chemokine (C-C motif) ligand 4 (MIP-1β/CCL4), RANTES/CCL5, Fractalkine/chemokine (C-X3-C motif) ligand 1 (CX3CL1), chemokine (C-X-C motif) ligand 9 (CXCL9), CXCL10, CXCL11 and monocyte chemoattractant protein 1/ chemokine (C-C motif) ligand 2 (MCP-1/CCL2), which are critical for function of monocytes and T-lymphocytes (43,46,48,52,63,76). Many of these have shown correlation to clinical parameters and disease severity. In all the chemokines measured, the concentrations of these molecules were higher in SS DED patients when compared with non-Sjögren’s (non-SS) DED patients.

#### Protein Biomarkers in Tears

The protein content in tears has been reported to be between 6 and 10 mg/ml and currently the tear proteome consists of about 1800-2000 proteins (59,77,78). The major proteins that have been reported in tears are lysozyme, Immunoglobulin A, lipocalin, albumin, lactoferrin and lipophilin which account for 70%-80% of the protein content. Mass spectrometry methods have also become sensitive enough to measure the proteome changes in the low abundant tear proteins. Lactoferrin and lysozyme are believed to be key proteins for anti-bacterial function in tears for the protection of ocular surface, while lipocalin is the major lipid binding protein in tears. Ohashi *et al*. have verified the change in tear levels of lactoferrin, EGF and aquaporin 5 proteins in a study of non-SS, SS and Stevens-Johnson syndrome patients using traditional immunoassays (79). Several proteins, namely, lysozyme-C, lipocalin 1, lactoferrin, lysozyme proline-rich protein 4 (LPRR4), lysozyme proline-rich protein 3 (LPRR3), nasopharyngeal carcinoma associated PRP4 and α-1 antitrypsin in addition to few other proteins were decreased in tears of disease patients (DED, SS, MGD) in multiple studies (22,23,80,81). Zhou *et al*., utilized i-TRAQ quantitative proteomics and identified four proteins that were decreased which include lipocalin-1 (LCN-1), prolactin-inducible-protein (PIP), lactoferrin and lysozyme (23). Similar results were reported using traditional immune assay or western blot methods. They also identified 6 proteins that were upregulated in tears from dry eye patients which include α-enolase, α-1-acid glycoprotein1 (AGP), S100A8/Calgranulin A, S100A9/Calgranulin B, S100A4 and S100A11 (Calgizzarin). The levels of α-enolase in tears, which is a key glycolytic enzyme, has been proposed to correctly identify a dry eye patient 85% of the time. AGP which is a member of the lipocalin family is a heavily glycosylated protein that plays an anti-inflammatory role. The S100 family of proteins are a family of calcium binding proteins that have been shown to have pro-inflammatory functions and have been identified as down-regulated in DED. Repeat studies that differentiated between different sub-groups of dry eye patients showed that dry eye subjects who had aqueous-deficient form of the disease showed distinct protein changes when compared to patients who had lipid-deficient or evaporative form of DED. Tear proteome and network analysis combined with ELISA validation studies have also led to proposal of tear biomarker panels with ability to discriminate between dry eye, MGD patients and control subjects (78,82). These unbiased MS/MS screens, combined with further validation of additional techniques have resulted in a list of potential biomarkers which have consistently been shown to be altered in multiple studies. The list includes lacritin, lactoferrin, lipocalin 1, PRR4, S100A8, S100A6, ceruloplasmin, Phospholipase A2, Cystatin S, lysozyme, secretoglobin family member 2A member 1, S100A9, and albumin (22,23,78,82–86). In addition to these extracellular proteins, several intracellular proteins such as Annexin A1 (ANXA1), Annexin A11 (ANXA11), aldehyde hydrogenase 3A1, clusterin, Glutathione-S-transferase P1, have also been shown to be deregulated in tears of dry eye patients (78,83,85). Given the variability in protein assessment using different methodologies across studies, grouping the proteomic biomarkers into an optimized panel of the most sensitive and repeatable proteins will offer a reliable test for ocular surface diseases. What is critical for the wide-spread use of tears as a source of biomarkers is the validation of this panel in independent studies across multiple cohorts of patients.

Other key proteins that have been evaluated in tears from dry eye patients are mucin 5 subtype AC (MUC5AC), and Cathepsin S (87,88). MUC5AC is a secretory member of the mucin family which are large high molecular weight glycoproteins playing an important role in lubrication, barrier formation and hydration functions of a mucosal surface. Several studies have shown decreased levels of MUC5AC in SS DED and non-SS DED and this has been shown to correlate with increased inflammation (89–92). Cathepsin S, a lysosomal cysteine endopeptidase involved in immune responses has been proposed as a candidate biomarker for SS based on the observation that Cathepsin S activity is significantly elevated (87,93). Neuromediators such as substance P, Nerve Growth Factor (NGF), Vasoactive Intestinal Peptide (VIP) and Calcitonin-Gene-Related Peptide (CGRP) have also been evaluated in tears (94). It has been demonstrated that NGF levels were elevated in DED while CGRP levels were decreased and NGF levels correlated directly where as CGRP levels correlated inversely to disease severity and that levels of neuropeptides were perturbed by contact lens wear (95,96).

It is evident that tear fluid analysis has become a key focus in ocular surface disease due to ease of access and advancement in analytical methodologies. Further development of guidelines of standardization of tear collection methods, processing and storage will enable comparison across studies and validate additional biomarkers.

## Conjunctiva

The conjunctiva is a part of the anterior segment of the eye. It is a thin, semi-transparent, highly vascularized, mucous secreting tissue that reflects forward on the eye at the fornix to cover the sclera and forms the inner lining of the upper and lower eyelids (4). Its primary function is in maintaining ocular surface homeostasis (97). In addition, it protects the soft tissues of the orbit and the eyelid, facilitates motion of the eyeball and eyelids, provides for the tear film’s aqueous and mucous layers, and provides a complex immunologic defense system.

Anatomically, the conjunctiva consists of three types, classified as palpebral, forniceal and bulbar conjunctiva. Histologically, it is composed of the epithelium containing stratified columnar cells, interspersed with mucin producing goblet cells and the substantia propria composed of connective tissue (98). The conjunctiva contains accessory lacrimal glands, lymphoid tissue, mast cells, and goblet cells. Goblet cells provide the mucinous component of the tear film through MUC5AC, gel-forming mucins that are central to many ocular surface disorders (99).

The lymphoid tissue associated with the conjunctiva, namely, the conjunctival associated lymphoid tissue (CALT) contains all the components of an immune response (100). In many ocular surface disorders, inflammation plays a critical role and the ocular mucosa is critical in modulating and resolving inflammation. Inflammatory cells such as eosinophils, basophils and mast cells normally are not present in the ocular epithelium (98). However, during inflammation, elevated levels of mucosal type mast cells are found in the epithelium. Proinflammatory modulators such as TNF-α, IL-6 and Interleukin-10 (IL-10) as well as various adhesion molecules, such as intercellular adhesion molecules (ICAM-1), in addition to various mononuclear cells, including Langerhans cells, cluster of differentiation 3 positive (CD3+) lymphocytes and cluster of differentiation 4 positive/cluster of differentiation 8 positive (CD4+/CD8+) lymphocytes are also found in the epithelial cell layer.

With advancement in sampling techniques, conjunctival tissue has become a valuable tool to evaluate biomarkers for multiple ocular disorders with minimal discomfort to the eye. The collection and analytical methodology followed by review of notable biomarkers in conjunctiva, is summarized below. Table II summarizes the key biomarkers in conjunctiva.

**Table II:** Summary of Key Biomarkers in Human Conjunctiva

Biomarker	Ocular disease	Function of the biomarker^a^	References
HLA-DR	DED	glycoprotein transmembrane complex, part of major histocompatibility complex class II; presents foreign peptides to the body’s immune system to trigger an immune response	(101,109–118,122,154,160,162)
ICAM-1	DED	Intercellular adhesion molecule; expressed by vascular endothelium, macrophages, and lymphocytes; key molecule involved in immune response	(112)
	SS		(120)
	OGVHD		(121,122)
Goblet Cells	SS	Secrete mucins onto the ocular surface	(126,132)
	Stevens-Johnson syndrome		(125)
	Graft-versus-host-disease		(127)
	DED		(125,128–131,133,134)
	Glaucoma		(135)
Mucins			
MUC5AC	AKC, SS, DED	Gel-forming glycoprotein; protects mucosa from infection and chemical damage	(137,142–145)
MUC16	AKC, DED	Mucin glycoproteins; acts as a lubricating barrier against foreign particles/infectious agents	(142–145)
Galectin-3	DED	Promotes formation of plasma membrane lattices	(147)
Inflammatory cytokines and chemokines			
IL-1α, IL-1β, IL-3, IL-6, IL-8	AKC, VKC, SS, GPC, TO related DE, DED, MGD, OGVHD	IL-1α: Proinflammatory cytokine involved in hematopoiesis IL-1β: Proinflammatory cytokine that promotes Th17 differentiation IL-3: Controls production, differentiation, and function of granulocytes and monocytes/macrophages IL-6: Inducer of the acute phase response and involved in the final differentiation of B cells IL-8: Directs the migration of neutrophils, basophils and T lymphocytes mediating innate immune and angiogenic response	(49,65,71,73,149–152,154,158,159)
TNF-α	DED, SS	Cytokine secreted by macrophages involved in inducing cell death of certain tumor lines; may stimulate cell proliferation/induce cell differentiation under different conditions	(49,73,149,150)
IFN-γ	DED, OGVHD	Immunoregulatory cytokine shown to play a role in TH 1-driven immune response at the ocular surface	(125,132,133,159)
TGF-β1	MGD, SS	Peptide; controls proliferation, differentiation, and other functions of T-cells, B-cells and myeloid cells	(49,152)
CCR5	DED	Receptor for CC chemokines;	(153)
CCL2, CXCL12, CCR2, CXCR4	DED	CCL2: Attractant for monocytes and basophils CXCL12: Chemoattractant on T-lymphocytes and monocytes; activates C-X-C chemokine receptor CCR2: receptor for CCL2, CCL7 and CCL13; signal transducer CXCR4: receptor for C-X-C chemokine and extracellular ubiquitin; increases intracellular calcium ions	(154)
Other potential biomarkers			
Histamine receptors (H1 and H4)	AKC, VKC	H1: Mediates contraction of smooth muscle and increases capillary permeability H4: Mediates inflammatory responses in allergic reactions	(155,156)
Eotaxin (CCL24)	AKC, VKC	Chemotactic for resting T-lymphocytes and eosinophils	(156,157)
PAX6	SS	Transcription factor involved in the devilment of eye, nose, CNS, and pancreas	(158)
SPRR1B	SS	Codes for envelope precursor proteins that are involved in barrier formation	(158)
NAMPT	OGVHD	Produces an enzyme involved in NAD synthesis	(159)
EGFR	OGVHD; DED	Protein; plays a vital role in cell proliferation during corneal wound healing	(159)
T lymphocytes CD4+, CD8+	DED, OGVHD	A group of white blood cells that develop from stem cells and mature in the thymus; involved in a variety of immune responses	(160–163)
HEL, 4-HNE, MDA	DED	HEL: An early product of lipid peroxidation 4-HNE: Major product formed by membrane lipid peroxidation; modulator of oxidative stress signaling MDA: Formed through the breakdown of unsaturated fats	(164)
Conjunctival proteome: ANXA1 and S100 proteins (S100A4, S100A6, S100A8, S100A9)	MGD, DED, Pterygium	ANXA1: Effector of glucocorticoid-mediated responses and regulator of inflammatory processes S100 proteins: Calcium binding protein; regulates inflammatory processes and immune responses	(165,166)

^a^function has been summarized based on information in references, and from website search: uniprot.org and google.com

### Collection of Conjunctival Cells and Analytical Methodology

Impression cytology (IC) is commonly used to collect superficial layers of conjunctival cells for analysis of biomarkers of ocular surface disorders. It is a well-established technique, that was developed at the end of the 1970s. It is easily repeatable, minimally invasive, and rapid collection technique for sampling superficial conjunctival epithelial cells in an almost painless manner (101,102). This technique uses absorbent filters which are applied to conjunctival surfaces and are made of cellulose acetate, polycarbonate, nitrocellulose or polyethersulfone (PES) (103). Recently, a single-use PES filter sampling device known as the Eyeprim device (Opia Technologies, France) has been introduced for IC sampling to better standardize the procedure. In a study in 20 healthy subjects comparing the amount of RNA recovered from conjunctival epithelial cells using the Eyeprim device and the conventional IC method, it was demonstrated that both methods provide similar RNA yield and result in comparable levels of discomfort without using anesthesia (104). Brush cytology is another technique used as an alternative to IC or can be used as complementary approach to collect conjunctival cells from a different region. A disposable brush is used, and an anesthetic may be applied prior to collection of cells from ocular surface (105). Brush cytology was found to be superior to IC in a 63-patient study that evaluated quantity and quality of cells harvested along with staining techniques, and cost (106). Multiple studies have shown utility of both brush cytology and IC in measurement of ocular surface biomarkers. Conjunctival samples are also collected by biopsies or excision of conjunctival tissue. This is a more invasive technique than above mentioned methods and less frequently adopted.

Upon sample collection, various analytical methodologies have been used to determine biomarkers in conjunctival cells. Microscopy, immunohistochemistry, flow cytometry and reverse transcriptase polymerase chain reaction (RT-PCR), are most widely used. Microscopy has been used to visualize cell morphology and count the goblet cell number in the conjunctival epithelium. For evaluation of goblet cell density, the IC membranes are fixed and stained using periodic acid Schiff (PAS) reagent and this technique has been well established. Immunohistochemistry and flow cytometry are techniques used to detect sub-clinical inflammation of ocular surface by measurement of inflammatory markers (105). Although, flow cytometry is a more standardized technique, independent of operator/lab dependent variability in measurements, there are certain limitations around sample integrity and IC storage conditions. Two-color flow cytometry is an advancement in the flow cytometry technique that uses double-immunostaining to investigate two cell surface markers on the same cell simultaneously. RT-PCR comprises of isolation of mRNA from IC samples and thus provides information about specific gene modulation on the ocular surface. Optimization of IC collection technique, RNA extraction and processing using an Illumina Human HT-12 BeadChip has led to successful transcriptome-wide gene expression analysis (107). Optimization steps led to an improved yield from analysis of 12 genes to 96 genes and then expression analysis of the entire human transcriptome.

*In vivo* confocal microscopy (IVMC) is a relatively novel technology for evaluating cellular changes at cornea and conjunctiva and has been used as a noninvasive diagnostic tool in several ocular surface disorders included DED (108). There are several challenges associated with this technique: from small fields of view to concerns around standardization of image acquisition, interpretation and quantification. Together with the high cost, this technology has not been widely deployed in clinical practice.

### Biomarkers in Conjunctiva

#### Human Leukocyte Antigen-D-Related

Human leukocyte antigen-D-related (HLA-DR) is a glycoprotein that is part of major histocompatibility complex class II cell surface receptor. It is normally expressed on the conjunctival epithelial cells, mostly in the immune-competent cells (109). Increased expression of HLA-DR has been associated with ocular surface diseases such as DED (109,110). Multiple studies have shown that expression of HLA-DR is significantly upregulated in patients with DED compared with normal eyes (110–113). HLA-DR is most commonly measured by flow cytometric analysis of conjunctival tissue samples taken by using IC. This technique for quantification of HLA-DR was initially demonstrated by Baudouin (114). A systematic assessment of sample stability, sensitivity, and reproducibility of IC as a technique to measure HLA-DR by flow cytometry was undertaken by Yafawi and group (101). This validation study demonstrated that increased expression of HLA-DR in patients with mild to severe DED, is sensitive enough biomarker to monitor for severity of disease. In addition, the study demonstrated high reproducibility in HLA-DR expression in all donors and highlighted certain limitations around sample integrity. HLA-DR expression was similar in samples from Day 1 and 10, but the expression decreased by day 14, suggesting loss of sample quality. Overall, the authors concluded that measurement of HLA-DR expression coupled with IC and flow cytometric analysis is a robust and reproducible assay, provided the IC samples are less than 10 days old.

HLA-DR has been widely used to monitor severity of disease, most commonly in DED and to evaluate potential of treatment effect during drug development. In a recent publication by Leonardi and group, the authors observed a clear relationship between HLA-DR expression and DED severity (115). Data from 2 Phase III studies was pooled and consisted of a 734 total DED patients, with 339 on vehicle and 395 on drug treatment (Cyclosporine cationic emulsion; CsA CE). Baseline HLA-DR expression values determined in 168 patients were directly proportional to corneal fluorescein staining (CFS) score, suggesting that disease severity correlated with increased ocular inflammation. In addition, they demonstrated the utility of this biomarker to monitor treatment response. At month 6, there was significant reduction in HLA-DR expression in CsA CE treated group *versus* vehicle (overall treatment difference: P=0.002). These data are consistent with other literature reports of reduction in HLA-DR expression by topical CsA (116). In a study comparing the efficacy of artificial tears *versus* 0.1% dexamethasone, reduced HLA-DR expression (P=0.01) was noted in patients treated with dexamethasone when compared to artificial tears (117). In another study in patients with DED, HLA-DR expression was determined in conjunctival cells by flow cytometry as a biomarker for treatment effect of topical ophthalmic tofacitinib at concentrations ranging from 0.0003% to 0.005% after 8 weeks of treatment (118). Even though there was no dose-dependent effect of tofacitinib observed in this study, a decrease in HLA-DR expression was observed in patients treated with tofacitinib 0.003% BID and 0.005% QD (67 % and 71 % of baseline, respectively) at week 8 when compared to patients treated with vehicle (133% of baseline). An active comparator, cyclosporine ophthalmic emulsion, 0.05% (Restasis, Allergan Inc., Irvine, CA), did not suppress HLA-DR expression in this study. This could be a consequence of fewer subjects and shorter duration in this study than previously reported duration of 3-6 months. In addition, the authors reported an association between the changes in HLA-DR expression and certain tear inflammation markers, such as IL-12p70 (r = 0.49) and IL-1β (r = 0.46). These studies demonstrate the potential of monitoring HLA-DR expression in conjunctival cells not only for evaluating disease severity but also in determining treatment effect and support its use as one of the established biomarkers in conjunctiva.

#### Intercellular Adhesion Molecule 1

Intercellular adhesion molecule 1 (ICAM-1), also known as cluster of differentiation 54 (CD54), is expressed on various cells such as endothelial cells, fibroblasts, leukocytes, keratinocytes and epithelial cells (119). It is upregulated in response to number of inflammatory mediators, including virus infection, proinflammatory cytokines, TNF-α and oxidative stress. Jones *et al*. used IC to demonstrate the upregulation of ICAM-1, among other inflammatory markers in the conjunctiva of patients with SS (120). In another study, Tsubota *et al*. used brush cytology and flow cytometry to quantitate HLA-DR and ICAM-1 expression in 28 dry eye patients (112). The authors reported increased expression of both these markers and in addition demonstrated that there was a good correlation between upregulation of ICAM-1 and HLA-DR in patients with DED. Aronni *et al*. evaluated ICAM-1 expression in patients with chronic graft *versus* host disease (cGVHD) who showed signs and symptoms of DED (121). IC samples collected from nasal and inferior bulbar conjunctiva showed increased expression in cGVHD eyes *versus* normal eyes. In addition, the authors reported an inverse correlation between ICAM-1 expression and goblet cell number, a marker of cell health. In a 32-patient clinical study, evaluating the safety and efficacy of topical tacrolimus for the treatment of OGVHD, changes in ICAM-1 expression in conjunctival epithelial cells were evaluated using IC (122). ICAM-1 expression decreased significantly (P=0.003) after 10-weeks of treatment with topical tacrolimus, when compared to baseline, thus demonstrating the utility of this biomarker in assessment of treatment effect. In the same study, expression of HLA-DR was evaluated, and tacrolimus significantly reduced HLA-DR expression at week 10 when compared to baseline (46% reduction; P=0.03). Thus, a combination of biomarkers can be used to increase confidence in drug effect in treatment of a disease.

#### Goblet Cells

Goblet cells are present within the conjunctival epithelial layer and are specialized cells that secrete mucins onto the ocular surface. The functions of the goblet cells include lubrication and surface wetting, maintenance of tear film and prevention of infection (123). Decrease in goblet cell density occurs in aqueous tear deficient dry eye and certain ocular surface inflammatory diseases, including SS, Stevens-Johnson syndrome, ocular mucous membrane pemphigoid, and OGVHD (124–126). When compared to normal subjects and patients with allogeneic hematopoietic stem cell transplantation without dry eye, patients with GVHD dry eye had decreased goblet cell numbers (127). In addition, the conjunctival inflammatory cells were significantly higher in these patients. Loss of conjunctival goblet cells results in decrease in mucin secretion and a damaged ocular surface. An increase in goblet cells may be an indicator of ‘healthy’ ocular surface and could be a biomarker for treatment effect. In patients with SS-KCS and non- Sjögren syndrome-associated keratoconjunctivitis sicca (NSS-KCS), conjunctival biopsy samples taken at baseline and after 6-month therapy with cyclosporine A (CsA) revealed a significant increase (P<0.05) in number of goblet cells at 6 months when compared to baseline (128). Several other studies reported increase in goblet cell numbers with topical CsA, suggesting the goblet cell number or density is a sensitive biomarker for detecting treatment effect in patients with DED or ocular inflammation (129,130). Another 32-patient study investigated the potential of a novel osmoprotectant, ISOMAR Eyes Plus in treatment of signs and symptoms of mild to moderate evaporative DED (131). Conjunctival IC showed a statistically significant increase (p <0.01) in GC density after 2 months of therapy (182.6 ± 28.6 cells/mm2) as compared to baseline (142.5 ± 25.6 cells/mm2). The biomarker response was associated with increase in tear stability and reduction in ocular surface damage. Evaluation in mouse model of dry eye indicated that goblet cells in conjunctiva modulate antigen distribution and antigen specific immune response, thereby contributing to ocular surface immune tolerance (124). Thus, loss or dysfunction of conjunctival goblet cells may be a significant factor contributing to loss of immune tolerance on ocular surface in DED. Goblet cell loss has been associated with increase in proinflammatory cytokines such as IFN-γ in DED (125,132,133). Thus, in a compromised or damaged ocular surface environment, increase in goblet cell number and reinstating its function, could be critical in restoring ocular surface homeostasis. Gumus *et al*. investigated the effects of the Allergan Intranasal Tear Neurostimulator (ITN) on conjunctival goblet cell function in a 15-participant (5 normal and 10 dry eyes), study (134). IC samples were taken at baseline and after each treatment: right eye samples were used for PAS staining and left eye samples were used for MUC5AC mucin immunostaining. The application of Allergan ITN stimulated goblet cell mucin secretion in addition to increasing tear production and goblet cell density, thereby resulting in novel treatment approach to DED. In 2018, Di Staso *et al*. reported results from a clinical study in 55 medically controlled glaucomatous patients, 17 DED patients and 17 healthy individuals aimed to evaluate goblet cell density using non-invasive in-vivo laser scanning confocal microscopy (135). The study revealed a significant reduction in goblet cell density in both glaucoma and DED groups compared to healthy controls (P<0.001) and the authors suggested that goblet cell reduction “may play a role in pathophysiology of the glaucoma-related disease of ocular surface”. In totality, these studies demonstrate the utility of measuring goblet cell density as an established biomarker indicative of ocular surface health.

#### Mucins

There are three types of mucins expressed in the conjunctival tissue, the secreted mucins expressed by the goblet cells, soluble mucins and the membrane-associated mucins, which are present in the conjunctival epithelial cells. Specifically, MUC1, MUC2, MUC4, MUC5AC, MUC7 and MUC16 mucin genes are present in the conjunctival epithelium (136,137). As demonstrated by *in situ* hybridization and immunofluorescence microscopy, the major gel-forming mucin MUC5AC is expressed by the goblet cells (138). The most studied membrane-associated mucins—MUCs 1, 4, and 16 are expressed in the conjunctival epithelial cells (138,139). MUC16 is also expressed by the goblet cells (140). The role of membrane-associated and secreted mucins in stabilization of tear film has been well established (141,142). In addition, these glycoproteins are responsible for lubrication of ocular surface, water retention and act as pathogen barriers. MUC7 is the soluble mucin, expressed on the ocular surface and its role is still unclear, but it may act as pathogen barrier (136). Investigating the changes in ocular mucins at the cellular level in conjunctival cells may help understand the pathogenesis of diseases such as atopic ocular allergies or DED and could act as important biomarkers of disease progression. In patients with severe AKC, conjunctival samples collected using IC and brush cytology, demonstrated that in eyes of patients with AKC, MUC16 mRNA expression was significantly upregulated and there was a simultaneous downregulation of MUC5AC mRNA expression, when compared to healthy control eyes (143). The authors postulate that the downregulation of MUC5AC could be a result of initial response of the ocular surface to inflammation in form of downregulation of epithelial mucins and loss of goblet cells, followed by upregulation of MUC16 to protect the ocular surface. In another study, IC samples collected from patients with SS, corroborate with the above findings (144). Using real-time RT-PCR, mucin gene expression profiles were quantified in the various IC samples and the data suggested that the expression of MUC5AC was significantly lower in 11 SS samples than in normal subjects. The levels of MUC5AC protein, measured in tear samples were also significantly reduced (P = 0.004), substantiating the data collected in the conjunctival epithelium. Thus, depletion of MUC5AC in tear fluids or ocular surface epithelium, could be a critical disease biomarker and indicator of compromised tear film stability and ocular surface health. Alterations in levels of MUC5AC and MUC16 have also been used to demonstrate modulation of disease following pharmacological intervention. Combined treatment of rebamipide (Mucosta® ophthalmic suspension UD 2%, Ostuka Pharmaceutical, Co., Ltd.) and steroid ophthalmic suspension was effective in increasing the expression of ocular surface mucins, MUC5AC and MUC16, from baseline in 2 DED patients, but data should be interpreted with caution due to the low number of subjects (145).

In DED, damage to cornea and conjunctiva is manifested as squamous metaplasia, characterized by loss of goblet cells, resulting in deficiency of mucins (140,146). Mucins, as mentioned earlier in this section, are the glycoproteins that form the glycocalyx, acting as a mucosal barrier, critical in ocular surface protection. An essential component of this glycocalyx barrier are the transmembrane mucins (MUCs), such as MUCs 1, 4 and 16, (147). It has been demonstrated that mucin distribution or mucin glycosylation in conjunctival epithelia changes with progression of disease (148). Evaluation of specific mucins along with their glycosylation pattern or associated glycans, could serve as important tools to measure disease progression and impact of therapeutic intervention. Galectin-3 is another such useful biomarker, which interacts with the transmembrane mucins at the apical glycocalyx (147). A study in 16 patients with DED, utilized IC samples to measure the expression of galectin-3, along with tear washes to examine whether it undergoes proteolytic degradation in tears (147). Interestingly, conjunctival expression of galectin-3 mRNA did not correlate with the increase in tear levels of galectin-3. The authors hypothesize that the discrepancy could be a result of disruption of the epithelial barrier causing alterations in transmembrane mucin glycosylation and loss of galectin-3 binding affinity, resulting in increased levels of cellular galectin-3 into the tear film in patients, when compared to normal subjects. Thus, galectin-3 could potentially be used as a novel biomarker in ocular surface disorders.

#### Proinflammatory Cytokines and Chemokines

Conjunctival epithelial cells play an active role in ocular surface defense and inflammation via release of pro-inflammatory cytokine and chemokine (chemotactic cytokines) mediators. Several studies have evaluated alterations in these mediators in tear fluid and these have been discussed in earlier section of this manuscript. This section outlines the utility of conjunctival cell sampling techniques to measure modulations in the pro-inflammatory mediator expression as biomarkers for disease or treatment effect.

In patients with AKC (n=10), VKC (n=10), and contact lens-associated giant papillary conjunctivitis (GPC, n=10), conjunctival biopsies were obtained under general anesthetic and expression of cytokines and chemokines (IL-3, IL-6, IL-8, GM-CSF, RANTES and TNF-α) was assessed using immunohistochemistry (149). The authors note that IL-8, IL-6, RANTES and TNF-α are localized to epithelial cells in normal conjunctiva and there was statistically increased expression of RANTES in all the allergic disorders compared to normal, along with increased expression of IL-8 in GPC when compared to normal, VKC and AKC. Normal conjunctival epithelial cells did not express granulocyte macrophage-colony stimulating factor (GM-CSF) and IL-3, but the GM-CSF was expressed by epithelial cells in all the disorders and IL-3 was expressed in VKC and AKC to equal degrees, but not in GPC. Overall, the study demonstrated that there are different cytokine profiles in the epithelial cells in the different clinical disorders and their measurement could result in improving our understanding of disease pathology.

Inflammation is one of the proposed mechanism for keratoconjunctivitis sicca or DED and elevated levels of inflammatory cytokines have been reported in conjunctival epithelium of dry eyes (49). In conjunctival cytology specimens taken from ten patients with SS_KCS and ten asymptomatic normal controls, significantly increased levels of IL-1α, IL-6, IL-8, TNF-α and transforming growth factor-beta1 (TGF-β1) were found in the conjunctival epithelium of SS patients when compared to controls (P < 0.05) and the concentration of IL-6 protein was significantly higher in SS conjunctiva samples (P= 0.012). Multiple other studies have explored alterations in pro-inflammatory cytokines and chemokines in DED patients and some of these results are discussed below (65,73,150). It has been shown that there is good correlation between the higher levels of these inflammatory mediators observed in conjunctiva and tear fluid. Massingale *et al*. reported increased mRNA expression of IL-1β, IL-6, IL-8, and TNF-α in conjunctival IC samples in dry eye patients as compared to normal controls and the fold increase (1.32 to 2.48) correlated well with the fold increase (1.55 to 2.90) of the cytokine tear levels (73). The authors postulate that the increased cytokine levels in tears of DED patients could be dependent on their expression in conjunctiva and that it may result in decreased tear production. In a study in diabetic patients with and without dry eye, and non-diabetic patients with dry eye, Zhang *et al*. reported significant increase in levels of IL-1β and TNF-α in biopsy samples collected from diabetic dry eye group and determined that the IL-1β and TNF-α positive cells were mainly localized in the basal layer indicating that inflammatory response may not be limited to the surface, but could be more serious in deeper layers of conjunctival epithelium (150). A study in patients with thyroid orbitopathy (TO) related dry eye, demonstrated increased levels of conjunctival cytokines IL-1α, IL-1β and IL-6 in IC samples using immunofluorescence (151) . IL-1β expression was noted to be significantly higher in patients than in control. This study was the first to evaluate cytokine expression in inflammation related to TO related dry eye.

In addition to increasing our understanding of the disease, modulation in cytokine or chemokine profile has also been explored to assess treatment effect. In MGD patients (n=16) treated with 1% azithromycin for 4 weeks, the expression levels of IL-1β and IL-8, were much higher (P < .001) than in healthy controls (152). The elevated levels of these mediators decreased after 4 weeks of azithromycin treatment, indicating that these could act as biomarkers for evaluation of treatment effect within a duration as short as 1 month. In this study, TGF-β1 expression was also monitored and was found to increase after treatment with azithromycin, indicating its role in clinical improvement of disease. It is important to note that the study did not include a control group and therefore, impact of factors independent of azithromycin treatment that could have influenced the expression of these mediators, could not be assessed.

Chemokine receptor up-regulation – Upregulation in expression of CCR5 has been observed in patients with both aqueous tear-deficient and evaporative forms of dry eye syndrome (153). The authors hypothesize that up-regulation of chemokine receptor may be secondary to ocular inflammation and in-turn may result in up-regulation of chemokine ligand. Another study in 32 patients with DED, examined the gene expression of chemokine ligands (CCL2 and CXCL12) and their respective receptors (CCR2 and CXCR4) in conjunctival IC samples (154). Expression of the CCL2, CXCR4 and CCR2 significantly increased in patients with DED as compared to control subjects and there was a trend for higher levels of CXCL12 but not statistically significant. Overall, the data strongly suggests that both chemokine receptors and their ligands are up-regulated in DED and could serve as useful biomarkers for disease modulation. These and other studies also indicate that specific chemokine receptors, such as CCR5, CCR2 and their ligands play a major role in modulation of inflammatory responses in DED and could serve as therapeutic targets.

#### Other Biomarkers in Conjunctiva

**Histamine** is known to play a critical role in ocular allergy by stimulating the expression of adhesion molecules and proinflammatory cytokines (155). Expression of histamine receptors (H1, H2, H3 and H4) was evaluated in conjunctival biopsy samples of 9 patients with active VKC and 6 healthy controls. Semi-quantitative RT-PCR demonstrated an over-expression of H1, H2, and H4 receptors in VKC *vs* control tissues, suggesting their important role in pathogenesis of allergic conjunctivitis. In particular, H4 receptors were highly expressed (5-fold more) in vernal tissues when compared to control tissues (155). Similar results were reported by Noriko *et al*. in a study in 19 AKC/VKC patients in which conjunctival samples were collected using modified IC (5 mm tip of Schirmer's test paper instead of a nitrocellulose membrane) in addition to scrapings of upper tarsal conjunctiva to obtain conjunctival smear specimens (156). The H4R expression was significantly increased in the active stage subgroup of AKC/VKC patients when compared to that in controls. This study also found strong correlation between H4R expression and eotaxin-2 levels, suggesting that H4R could be a useful biomarker for inflammation associated with eosinophilic infiltration of ocular surface.

**Eotaxin** is a member of the CC chemokine family and is divided into three subfamilies, namely, CCL11/eotaxin-1, CCL24/eotaxin-2, and CCL26/eotaxin-3 (157). It has been reported that increased eotaxin-2 levels in tears and higher expression of CCL24 (eotaxin-2) mRNA on the ocular surface is common in ocular allergies. In a study in 18 patients with VKC/AKC, the eotaxin-2 expression levels were significantly higher in active stage subgroup of the AKC/VKC group, when compared to those in the stable stage subgroup of AKC/VKC group and the control group (157). In addition, in patients, clinical scores were significantly correlated with the levels of eotaxin-2 mRNA expression on the ocular surface (ρ = 0.795, P < 0.01,), indicating that monitoring of the expression levels of eotaxin-2 mRNA in modified IC samples may provide a useful index of disease exacerbation and therapeutic response.

**Paired-box protein 6 (PAX6)** is another potential biomarker that has been evaluated as a marker of ocular surface damage, using IC collection technique. PAX6 is commonly expressed throughout the entire ocular surface epithelium i.e., from cornea, limbus to conjunctiva (158). McNamara *et al*. demonstrated that PAX6 expression was significantly reduced in SS patients and highly correlated with ocular staining score (158). In the same study, small proline-rich protein (SPRR1B), was associated with ocular damage and significantly elevated in SS patients. Both, PAX6 and SPRR1B expression, could serve as robust predictors of disease severity, resulting in ocular surface damage.

**NAMPT** (nicotinamide phosphoribosyltransferase, also called visfatin), is a proinflammatory cytokine that is poorly understood and has not been described extensively in ocular disease pathology (159). It inhibits neutrophil apoptosis and promotes B cell maturation. In a study in 20 OGVHD patients and 14 healthy controls, expression of 84 genes was determined in IC samples and among these, NAMPT was identified as one of the 4 genes that had the greatest potential as diagnostic biomarker that was clinically relevant. The other 3 genes identified were IL-6, IL-9 and epidermal growth factor receptor (EGFR). Higher expression levels of IL-6, IL-9, and NAMPT correlated with lower tear production, greater ocular surface damage and tear film instability, whereas decreased EGFR expression was associated with redness and ocular surface damage. Interestingly, EGFR gene was the only gene that was downregulated in OGVHD patients (159). There is evidence to suggest that in comparison to healthy controls, the soluble EGFR levels are significantly greater in DED patient tear samples (48).

**T lymphocytes** are known to play an active role in inflammation of anterior surface of the eye. In conjunctival biopsy specimens obtained from patients with DED, immunohistochemistry has shown an infiltrate of T cells (CD3+, CD4+) in the connective tissue component of the conjunctiva known as substantia propria (160). In 21 patients with DED, samples collected by IC demonstrated a significant difference in the CD4+/CD8+ ratio in dry eye group with respect to control. This study also showed a novel method to preserve the IC samples such that it significantly increased the number of cells harvested from the filter paper (160). Another study in patients with evaporative type DED, demonstrated a significant modification in CD4+/CD8+ ratio with corticosteroid treatment in addition to lid hygiene and were negatively correlated to tear lysozyme levels (161). The authors postulate that the associations between inflammatory mediators and clinical endpoints provide evidence that these biomarkers are useful in diagnosis of DED.

The only objective diagnostic test available for clinical diagnosis of ocular cGVHD is the detection of differentiated CD4+ and CD8+ lymphocytes in conjunctival biopsies (162). Unfortunately, biopsies are invasive and may severely impair the patient. A study in 18 patients with ocular cGVHD demonstrated that the detection of CD8+ lymphocytes using IC was frequently correlated with ocular cGVHD and could be used as a less invasive strategy for diagnosing cGVHD status (162). A more recent evaluation of T cell subsets (CD4+ and CD8+ naïve, T_CM_ and T_EM_) at the ocular surface, utilizing IC technique, has suggested the possibility of using T-cell immune signatures and associated clinical findings as a tool to stratify patients during clinical trials evaluating immunomodulators (163).

**Lipid peroxidation markers**: It is known that oxidative stress plays a critical role in cellular injury, resulting in ocular surface disorders. Measurement of the end products of lipid peroxidation is one of the widely accepted approaches to detect oxidative damage (164) . Among the oxidative markers, hexanoyl-lysine (HEL) is an early product of the lipid peroxidation process, whereas 4-hydroxy-2-nonenal (4-HNE) and malondialdehyde (MDA) are late-phase markers. The expression of these markers (HEL, 4-HNE, and MDA) in the conjunctival IC samples of 44 patients with n-SS dry eye and 33 control subjects, was evaluated using immunohistochemistry (164). The expression of 4-HNE and MDA was higher in the conjunctival epithelium in DED patients when compared to controls and these results correlated with increased levels of these markers in tear fluid and ocular surface parameters including Schirmer test and goblet cell density. The data suggests potential utility of these markers in determining the severity of DED.

**Conjunctival epithelium proteome**: Several proteomic studies in conjunctival tissue have determined inflammatory and apoptotic biomarkers but there remains a need to understand the role of conjunctival proteins in disease pathophysiology (165). Acquiring sufficient conjunctival protein material from IC sampling appears to be challenging. To overcome these limitations, Soria *et al* implemented a novel technology, two-dimensional difference gel electrophoresis (2D-DIGE) for proteomic analysis of IC samples collected from patients with MGD (n=41), DED (n=43), and healthy subjects (n=42) (165). The most highly expressed protein markers were annexin A1 (ANXA1), calcium activated signaling protein S100A8 (S100A8) and protein S100A4 (S100A4), and these markers were further validated and confirmed using dot blot assays. Both techniques revealed significantly elevated levels of ANXA1, S100A8, and S100A4 in the DED and MGD patients when compared to control subjects. In addition, Pearson correlation analysis demonstrated a significant correlation between these 3 biomarkers and clinical parameters such as Schirmer, TBUT, and SM. This study is the first to focus on high- throughput proteomic analysis of conjunctival IC samples and has demonstrated its utility in disease stratification and monitoring of treatment response.

S100 proteins are low molecular weight, calcium-binding proteins that have been shown to be present in both normal conjunctiva and pterygial epithelium (166). Pterygium is characterized by epithelial and fibrovascular overgrowth of conjunctiva over cornea. Higher expression levels of S100A6, S100A8, and S100A9 were observed in the pterygium tissue when compared to normal conjunctiva and it has been postulated that these proteins may be associated with the pterygium formation (166).

Thus, a large variety of biomarkers have been identified in the conjunctival tissue which has extended our knowledge of key ocular surface disorders and has allowed for assessment of response to treatment. Utilization of IC sampling technique has further enhanced evaluation of biomarkers in this tissue making it safe and easily accessible. As with tear fluid analysis, improved sensitivities in protein-based and gene-based analysis techniques, could result in identification of more specific biomarkers in conjunctiva in future.

## Aqueous Humor

According to the Vision Eye Institute, aqueous humor (AH) is defined as a thin, clear fluid filling the space in the anterior compartment of the eye between the lens and the cornea. It is composed primarily of water (99.9%) and trace amounts of sugars, vitamins, proteins and other nutrients as well as growth factors and cytokines. In addition to maintenance of intraocular pressure (IOP), the AH serves multiple other functions in support of ocular health. The AH also provides nutritional support to the cornea and lens in addition to physical support in maintaining the shape of the eye. Proper fluid resistance is controlled by the interaction of multiple structures in the eye which include but are not limited to ciliary muscles, Schlemm’s canal (SC), the trabecular meshwork (TM), and aqueous veins. AH drains from the eye via one of two passive pathways – the traditional TM pathway and the uveoscleral, or unconventional pathway. The traditional pathway involves contraction of the ciliary muscle which causes the TM to expand allowing for AH outflow through the TM. The uveoscleral route drains AH through the uvea, ciliary body and muscle into the choroid and sclera (167–175).

Due to its proximity to the site of pathogenesis in glaucoma, the discovery and detection of biomarkers in the AH can provide valuable information for the development of future antiglaucoma therapeutics. Biomarkers in the AH have also been discussed and used in other ocular diseases such as diabetic retinopathy (176–178). Associations have also been made between biomarkers in glaucoma and non-ocular diseases such as Alzheimers (179). For the purposes of this review, we will focus on the utility of glaucoma biomarkers in the AH.

An effective biomarker that can be detected in the AH of glaucoma patients would have multiple benefits and poses a unique opportunity to both monitor disease in patients as well as guide development of new therapeutics. For instance, biomarkers could be potentially useful in the event of ocular asymmetry as is often observed in exfoliation syndrome (ES) or pseudo-exfoliation (PEX) glaucoma. Two thirds of patients present in one eye, but half of those patients will eventually display symptoms in the contralateral eye within 15 years. A biomarker to help guide physicians to diagnose earlier or monitor a patient’s response to treatment could be particularly useful in these cases (180). In normotensive glaucoma, where optic neuropathy progresses in absence of elevated IOP, a biomarker indicative of optic nerve damage or health of anterior segment is even more desirable. Sections below review the biomarkers in AH along with the advances in the analytical methods. Table III summarizes the key biomarkers in AH.

**Table III:** Summary of Key Biomarkers in Human Aqueous Humor

Biomarker	Ocular disease	Function of the biomarker^a^	References
Genetic biomarkers			
LOXL1	Normotensive glaucoma	Encoded protein essential to biogenesis of connective tissue	(180)
	Exfoliation syndrome/glaucoma		(191)
	Pseudoexfoliation glaucoma		(190)
ATX	Glaucoma	Secretory protein; involved in the regulation of IOP	(194,195)
Myocilin	Glaucoma	Believed to have role in cytoskeletal function; mutation of gene is a major cause of glaucoma	(196,197)
Growth factors			
TGF-β	POAG	Peptide; controls proliferation, differentiation, and other functions of T-cells, B-cells and myeloid cells	(188,192,198–203,232,233)
HGF, TGF-β2	Glaucoma	HGF: Cellular growth, motility, and morphogenic factor; affects T-cells TGF-β2: Cytokine; suppresses the effects of interleukin	(231)
Inflammatory mediators			
TNF-α	POAG	Cytokine secreted by macrophages involved in inducing cell death of certain tumor lines; may stimulate cell proliferation/induce cell differentiation under different conditions	(207,208)
IL-1α, IL-6, IL-8	POAG	IL-1α: Proinflammatory cytokine involved in hematopoiesis IL-6: Inducer of the acute phase response and involved in the final differentiation of B cells IL-8: Directs the migration of neutrophils, basophils and T lymphocytes mediating innate immune and angiogenic response	(209)
ELAM-1 (E-Selectin)	Glaucoma	Selectin cell adhesion molecule; expressed only on activated endothelial cells	(209,211,212)
APO AI, APO CIII, APO E, TTR, α2-macroglobulin, Cystatin-C	POAG	APO AI: Protein; enables efflux of fat molecules from tissues to the liver APO CIII: Protein; inhibits lipase activity APO E: Protein involved in aggregation/clearance of amyloid-β TTR: Thyroid hormone binding protein; transports thyroxine from the bloodstream to the brain α-macroglobulin: Antiprotease Cystatin-C: Extracellular inhibitor of cysteine proteases	(179)
sNCAM, VCAM-1, Cathepsin D	POAG	sNCAM: Membrane-bound glycoprotein; facilitates cell-cell adhesion VCAM-1: Vital protein in cell-cell recognition Cathepsin D: Acid protease; causes intracellular protein breakdown	(206,210)
Biomarkers for vascular tone and architecture			
BNP, ANP	POAG	Causes vasodilation, natriuresis, inhibition of the renin-angiotensin-aldosterone and sympathetic nervous system	(218,219)
Oxidative Stress biomarkers			
SOD	Glaucoma; POAG	Enzyme; catalyzes superoxide radicals	(220–223)
GPX	POAG, PACG	Protects against oxidative damage	(220)
Proteomic biomarkers			
CD44S	PACG	Mediator of cell-cell and cell-matrix interactions	(229)
NMDA receptor	POAG	Receptor of homocysteine that can cause cell death	(229,230)

^a^function has been summarized based on information in references, and from website search: uniprot.org and google.com

### Collection of Aqueous Humor and Analytical Methodology

AH samples are collected via aqueous tap in patients undergoing cataract surgery, trabeculectomy, phacoemulsification or from post-mortem eyes. Collection is generally performed as an outpatient procedure with only local anesthesia. However, in children or uncooperative patients, sedatives may also be used. Collection volumes are relatively small and range from 100-250 μL. The total volume of AH in the anterior chamber and the rate of turnover (estimated to be ~2.5 μl/min) needs to be considered when determining collection volume and frequency of aqueous tap (176,181–185). Although potentially valuable information can be derived from these samples, there are also potential risks associated with collection of the samples themselves. Methods of collection are highly invasive and put patients at risk for additional damage to cornea and lens. Contact with other structures in the eye during the collection process can also contaminate samples with non-AH proteins. Samples can be obtained from post-mortem eyes but will have significantly different profiles than those collected from live patients due to accumulation of metabolic waste and other uncontrolled post-mortem processes. Therefore, samples collected from live patients are deemed most useful.

Mutiplex bead immunoassays like Luminex are ideal for analyzing small volume samples such as tears and likewise have proven effective in measuring cytokine levels in AH. Advancements in proteomics, genomic, and metabolic techniques have increased sensitivity and detection of products that may serve as potential biomarkers. Sensitivity of these techniques is especially important due to the low volume of AH samples (180). Additionally, basal levels of proteins in the AH are relatively low, containing only 120-500 ng/μL of protein which is thought to decrease with age. Multiple proteins have been evaluated in AH samples and advancements in mass spectrometry have substantially improved detection methods in less than a decade. Chowdhury *et al*., identified 676 proteins in human AH using nanoflow liquid chromatography electrospray ionization tandem mass spectrometry (nano-L-ESI-MS/MS), (171). Murthy *et al*. were able to identify 763 proteins using a combination of in-gel digestion, in-solution digestion followed by basic pH-RPLC coupled with mass spectrometry, (186). More recently, Adav *et al*. used high-performance LC-MS/MS to identify 865 proteins in AH in patients with Primary Open-Angle Glaucoma (POAG) with a false discovery rate of less than 1% (187). Another approach, RNA sequencing (RNA-Seq), allows for rapid profiling and thorough investigation of the transcriptome and offers numerous benefits compared to other analytical methods. It’s ability to detect novel transcripts and single nucleotide variants, among other endpoints, is a valuable tool for analysis of human AH samples with potentially low-abundance transcripts. Furthermore, with next-generation sequencing (NGS) techniques miRNome analysis of small samples can be performed which avoids some of the limitations of hybridization-based detection methods (188). Gene array analysis is an additional powerful technique for comparing gene expression profiles in AH. Using a miRNA analysis system (e.g. Toray Industries) and AH miRNA samples hybridized to 3D-Gene human miRNA chips, miRNA gene expression data can be obtained. Following identification of significantly changed miRNAs, bioinformatical analysis can be performed to predict the molecular targets. Tanaka *et al*. performed the first study to identify candidate biomarker miRNAs in AH of glaucoma patients. Ingenuity Pathway Analysis (IPA) was also employed to link those miRNAs to molecular pathways and targets (189).

### Biomarkers in Aqueous Humor

#### Genetic Biomarkers

The lysyl oxidase-like 1 (LOXL1) gene, identified as a genetic risk factor for exfoliation glaucoma or PEX is a cross-linking enzyme involved in extracellular matrix metabolism. Increased levels of LOXL1 are involved in the formation of abnormal fiber aggregates in exfoliation glaucoma or PEX (190,191). LOXL1 also interacts with TGF-β1 in the formation of elastic fibers (192). LOXL1 positive deposits have been found in outflow structures and are thought to contribute to elevated IOP and optic nerve damage (193). Autotaxin (ATX), a secretory protein is a source for extracellular lysophosphatidic acid (LPA). Data suggests a connection between the ATX-LPA pathway and elevated IOP in glaucoma. Elevated levels of ATX been observed in the AH of glaucoma patients (194,195). Elevated levels of ATX and LPA have also been significantly correlated with IOP and glaucoma subtype (194). Myocilin has also been identified as another potential genetic marker of glaucoma in AH samples. Myocilin, a glaucoma associated protein, has been found elevated in ocular tissue, in AH of animal models of glaucoma and in humans (196). Howell *et al*. evaluated AH samples from glaucoma patients and found, via Western Blot, that myocilin was increased in 70% of the evaluated POAG patients (197).

#### Growth Factors

Elevated levels of pro-fibrotic growth factors, such as TGF-β have been reported by many sources to be significantly increased in the AH of POAG patients (185,198–203). Multiple isoforms of TGF-β have been described in the literature but TGF-β2 is considered the main isoform of ocular tissue. TGF-β2 is synthesized in the anterior segment of the eye and is considered a multifunctional growth factor. Relevant to glaucoma, TGF-β2 promotes extracellular matrix production and decreases cell proliferation. A meta-analysis of eight published studies reporting elevated levels of TGF-β2 in AH of multiple sub-types of glaucoma, clearly demonstrated that TGF-β2 is elevated in open-angle glaucoma. Differences in increased levels of total and active TGF-β2 were dependent on the type of glaucoma (198).

#### Inflammatory Mediators

Neuroinflammation and pro-inflammatory cytokines such as TNF-α have been implicated in glaucoma (204–206). Several studies using ELISA and single-plex bead immunoassay techniques have shown increases in TNF-α in the AH of glaucoma patients when compared to controls. Sawada *et al*. reported slight increases in TNF-α in the AH of POAG and normotensive patients as well significant increases in patients with exfoliation glaucoma (207). A subsequently reported study found >3-fold differences in TNF-α levels in POAG patients compared to controls (208). Other cytokines such as IL-1α, Il-6, and IL-8 have also been identified to be increased in AH of POAG patients (209).

Markers identified in other neurodegenerative diseases, such as Alzheimer’s disease, have similarly been explored in the AH of glaucoma patients. Inoue *et al*. has shown that proteins such as apolipoprotein (APO) AI, APO CIII, APO E, transthyretin (TTR), α2-macroglobulin and Cystatin-C, which are known to be elevated in Alzheimer’s disease, are also increased in the AH of POAG patients (179). Zhang *et al*. analyzed AH of POAG patients using the Luminex Human Neurodegenerative Disease Panel 3 and found that soluble neural cell adhesion molecule (sNCAM), soluble vascular cell adhesion molecule-1 (sVCAM-1) and cathepsin D, levels were significantly increased in glaucoma patients compared to controls (206). Cathepsin D is a lysosomal aspartic protease that plays a role in cell homeostasis and cell death (210). Capthesin D has also been found to be elevated in the cerebral spinal fluid of Alzheimer’s disease patients demonstrating its broader role in neurodegeneration. VCAM-1, can be induced by TNF-α, and is a marker of vascular remodeling, endothelial activation and dysfunction, and leukocyte infiltration. TNF-α has also been shown to be elevated in the AH of POAG patients. NCAM is an additional cell adhesion molecule that has been implicated in multiple neurological and neurodegenerative disorders (204,206,208,210). These results may be mirroring damage occurring in the TM.

Endothelial leukocyte adhesion molecule-1 (ELAM-1), sometimes referred to as E-Selectin, is an endothelial cell surface glycoprotein subjected to activation by cytokines and is responsible for the adhesion of inflammatory cells such neutrophils, monocytes, and T-cells (211). It also happens to be one of the first markers established for atherosclerotic plaques in vessels and interestingly has been found to be present and activated in the TM cells from glaucoma patients (212). AH samples collected from glaucoma patients and analyzed by antibody microarrays have found ELAM-1 to be significantly elevated compared to controls (209).

#### Biomarkers Related to Vascular Tone and Architecture

Potential biomarkers have been proposed related to vascular tone and architecture in glaucoma. Altered levels have been observed in serum but very few have been identified in AH. Proposed biomarkers found in the AH include: cyclic guanosine monophosphate (cGMP), nitric oxide (NO), brain and atrial natriurectic peptide (BNP and ANP respectively). It is well documented that NO and its second messenger cGMP are involved in homeostasis of AH dynamics and IOP (213,214). NO and cGMP have been found to be significantly decreased in AH of patients with POAG but contrasting outcomes have been observed between the different subtypes of glaucoma as well as in other structures (214–216). While under debate, it has been proposed that these differences may be reflective of the different pathology expressed between glaucoma subtypes (217). BNP and ANP are cyclic endopeptidases that are involved in water excretion and vasodilation. ANP is considered a biomarker for cardiac hypertrophy but has also been detected in AH. A fragment of the ANP prohormone was recently detected at much higher levels in the AH of POAG patients undergoing trabeculectomy compared to control patients undergoing cataract surgery (218,219).

#### Oxidative Stress Markers

Oxidative stress and antioxidant status have been implicated in multiple ocular diseases including glaucoma. Once oxidative stress occurs, reactive oxygen species (ROS) levels exceed the antioxidant defense capacity. This imbalance is thought to disrupt proper function of the TM. The TM is particularly sensitive to oxidative stress due to its innate defense mechanisms meant to protect against ROS. As a result, perturbations of this system likely play a significant role in the pathogenesis of glaucoma (220–223). Multiple markers of oxidative stress that can be detected in AH have been reported in the literature and linked to glaucoma. Data suggests that oxidative stress induces antioxidant enzymes that in turn may facilitate decreased reactive antioxidant potential leading to glaucomatous damage. Moreover, products such as glutathione peroxidase (GPX), superoxide dismutase (SOD) and malondialdehyde (MDA) have been observed at irregular levels in the AH of patients with POAG. SOD is a key antioxidant enzyme involved in the metabolism of oxygen-free radicals and prevents the formation of other ROS (221,223). In a case control study which sampled AH from patients undergoing cataract surgery, patients with glaucoma presented with 57% higher SOD activity compared to the non-glaucomatous patients also undergoing cataract surgery (221). Goyal *et al*. also observed significant increases is SOD in POAG (46.19 ± 6.79 U/mL), and primary angle-closure glaucoma (PACG; 44.38 ± 6.47 U/mL) compared to cataract controls (21.70 ± 4.93 U/mL; p<0.0001) (222). GPX was also elevated in the AH of POAG (20.58 ± 6.79 U/mL) and PACG eyes (19.27 ±3.84 U/mL) compared to cataract controls (8.17 ± 2.97 U/mL; p<0.0001).

Antioxidants such as Vitamin C and E, also found in the AH, exhibit protective roles against free radical damage and lipid peroxidation. In addition, the synthesis of extracellular matrix molecules such as collagen, elastin, laminin and glycosaminoglycans is impacted by Vitamin C. Compromising this system and altering levels of Vitamin C may have important implications for the function of the TM. Both have been detected at altered levels in the AH of POAG, PACG and ES patients. Vitamin E plays an important role in the maintenance levels of peroxide (H_2_0_2_) in the AH. Vitamin E deficiencies can lead to dysfunctional cells in the TM, damage to the lamina cribrosa and axons of the optic nerve. Together, these markers and changes observed in the AH may be early indicators of future damage in glaucoma patients or even at-risk populations (222,224–226).

Benoist d’Azy conducted a systematic review and meta-analysis of 22 case control studies which evaluated oxidative and antioxidative markers in AH and serum samples of glaucoma patients (220). An overall increase in oxidative stress markers was observed in glaucoma patients in both types of matrices. However, a disconnect was observed between serum and AH with measures of antioxidative stress markers SOD (effect size = 3.53; 95% CI 1.20-5.85) and GPX (effect size = 6.60, 95% CI 3.88 -9.31) which were elevated in AH but not serum. It is hypothesized that the increase in antioxidant markers may reflect a local, compensatory response in the eye against oxidative stress.

#### Proteomics

Characterization of the AH proteome provides insights into anterior segment homeostasis and may reflect damage in the TM (171,185,227,228). Proteomic analysis of AH samples from patients suffering from PACG have shown an enrichment of atypical collagens and fibronectins and higher levels of soluble CD44S compared to healthy adults (176,229). Homocysteine, a neurotoxin that induces apoptotic cell death of retinal ganglion cells (RGCs) via the N-methyl-D-aspartate (NMDA) receptor and may play a role in optic nerve damage in POAG, has also been found to be elevated in glaucoma patients using proteomic methods (230). Using ELISA, multiple growth factors including hepatocyte growth factor (HGF) and TGF-β2 have been shown to be increased in the AH of patients suffering from glaucoma (190,231–233).

Izzotti *et al*. utilized cyanine-labeled protein samples from the AH of POAG patients which were then hybridized with antibody arrays to identify 31 proteins with a greater than 2-fold variance compared to controls (234). This work has provided insight into pathways and mechanisms of POAG such as mitochondrial dependent and independent apoptotic mechanisms, oxidative stress, and neural survival. Furthermore, it provides additional evidence and support for the proteomic analysis of AH samples as a tool for investigating mechanisms of glaucoma

#### Surrogate Endpoints

Not all biomarkers require a biochemical assay of bodily fluid; some endpoints can be measured via non-invasive, physical measurements. These types of endpoints are often called surrogate biomarkers, or surrogate endpoints. The biomarker definitions working group, convened by NIH, defined a surrogate endpoint as “*a biomarker that is intended to substitute for a clinical endpoint”* and stated that *“*a surrogate endpoint is expected to predict clinical benefit (or harm or lack of benefit or harm) based on epidemiologic, therapeutic, pathophysiologic, or other scientific evidence” (5). A classic example of such a biomarker used in ophthalmology is the use and measurement of intraocular pressure (IOP). Elevated IOP is the biggest risk factor for glaucoma and optic nerve damage. Antiglaucoma therapeutics have been approved based on their ability to reduce IOP and not necessarily their ability to prevent further damage to the optic nerve and RGCs. Currently, it is the only surrogate endpoint used by the FDA to evaluate drug treatment in glaucoma patients with ocular hypertension (235). Although IOP measurement is an FDA accepted surrogate endpoint, it is not a relevant endpoint for all forms of glaucoma (e.g. normal tension glaucoma). Likewise, an antiglaucoma agent may reduce IOP but not inhibit progressive vision loss. Structural endpoints such as optic nerve head (ONH) rim width, area, and retinal nerve fiber layer thickness (RNFLT) using OCT in glaucoma patients are also valuable surrogate endpoints (5,236–238).

As with all endpoints, there are advantages and disadvantages of using surrogate endpoints. The use of surrogate endpoints in clinical trials may reduce the duration of the study, as well as sample sizes, which in turn can result in significant cost savings. However, the use of surrogate endpoints can also be damaging and lead to misinterpretation of findings if endpoints aren’t properly validated (238–241).

The possibility of a biomarker measured in the AH meeting all these criteria and replacing IOP measurement, is highly unlikely. Especially considering the invasiveness of AH taps compared to IOP measurement. However, merging multiple biomarker and surrogate endpoints may be a better assessment of drug efficacy and the patient’s overall prognosis.

## Vitreous

The vitreous functions in maintaining the eye’s spherical shape and pressure, protecting the eye from physical injury, and keeping the retina in place. It is the largest chamber of the eye and is located in the posterior segment between the lens and retina. The vitreous is a clear gelatinous extracellular matrix that is comprised of mostly water (99%) and a meshwork of fine collagen fibrils embedded with dissolved hyaluronan molecules, inorganic salts, and lipids (242,243). The spacing of the fine collagen fibrils is maintained by macromolecules such as opticin and proteoglycans and the hyaluronan is thought to increase the mechanical resilience of the gel (244). The vitreous also contains proteins such as albumin, globulins, coagulation proteins, and complement factors that have accumulated from local secretion, filtration of blood, or diffusion from surrounding tissue and vasculature (245,246). It’s anatomical position near the retina makes it an ideal compartment to sample for biochemical and pathophysiological changes when there is retinal or vitreoretinal disease states including proliferative diabetic retinopathy (PDR), DME, and AMD. The sampling access to the vitreous grants the potential of assessing pathophysiologic changes to molecular biomarkers as guides to determining ocular disease severity, patient population selection, and/or evaluation of treatment effect for current or future therapeutics. This section includes a summary of the current vitreous collection and analytical methodologies, followed by a review of both extensively studied and newly proposed vitreous biomarkers. Table IV summarizes the key biomarkers in vitreous.

**Table IV:** Summary of Key Biomarkers in Vitreous

Biomarker	Ocular disease	Function of the biomarker^a^	References
Angiogenic and Anti-angiogenic markers			
VEGF	DR, PDR, NVG, DM	Induces endothelial cell proliferation, promotes cell migration, inhibits apoptosis, induces permeabilization of blood vessels	(253,255–259,261,262,264,267,268)
PIGF	PDR, NVG	Involved in glycosylphosphatidylinositol-anchor biosynthesis	(260,261)
sVEGFR	PDR, AMD, Neovascular glaucoma	Regulates angiogenesis, vascular development, vascular permeability, and embryonic hematopoiesis	(261,262)
PDGF (-AA, -AB, -BB)	NPDR, PDR	Regulator of embryonic development, cell proliferation, cell migration, survival, and chemotaxis	(264,265,268)
PEDF	PDR, AMD	Anti-angiogenic; suppresses retinal neovascularization and endothelial cell proliferation	(261,266–268)
Inflammatory mediators and neurotrophins			
IL-1β, IL-6, IL-8, and TNF-α	PDR, DR, DM, NVG	IL-1β: Proinflammatory cytokine that promotes Th17 differentiation IL-6: Inducer of the acute phase response and involved in the final differentiation of B cells IL-8: Directs the migration of neutrophils, basophils and T lymphocytes mediating innate immune and angiogenic response TNF-α: Cytokine secreted by macrophages involved in inducing cell death of certain tumor lines; may stimulate cell proliferation/induce cell differentiation under different conditions	(258,259,267–270)
NGF, BDNF, NT-3, NT-4, CNTF, GDNF	PDR, NPDR	NGF: Activator of cellular signaling cascades through tyrosine kinase receptors BDNF: Neurotrophic growth factor; encourages growth and differentiation of new neurons and synapses NT-3: Receptor for BDNF; involved in neuron survival, proliferation, and migration NT-4: Survival factor for peripheral sensory sympathetic neurons CNTF: Prevents degeneration of motor axons GDNF: Promotes survival and differentiation of dopaminergic neurons	(269)
TAT	RRD, PDR	Regulates blood coagulation cascade	(270)
Hemodynamic markers			
NO, ET-1	PDR, NPDR	NO: Creates loss of autoregulation in diabetic retinas ET-1: Vasodilator; produced by vascular endothelial cells	(268,274)
Acute Phase factors			
serum amyloid P, procalcitonin, ferritin, fibronectin, fibrinogen (α, β, γ chain)	DME, PDR	Serum amyloid P: Contributor to the pathogenesis of amyloidosis, including Alzheimer’s Procalcitonin: Acute phase reactant; precursor for calcitonin Ferritin: intracellular protein involved in storage and release of iron Fibronectin: Glycoprotein; binds to integrins on membrane surfaces; plays role in cell adhesion, growth, migration, and differentiation Fibrinogen: Occludes blood vessels to prevent excessive bleeding; binds and reduces the activity of thrombin	(275,276)
Pentraxin-3	PDR	Released by various lymphocytes in response to primary inflammatory signals; promotes fibrocyte differentiation and inflammation activation	(277)
Advanced glycation end products			
glycer-AGEs (TAGEs); sRAGE	PDR	TAGEs: Glycated proteins or lipids that worsen many degenerative diseases sRAGE: Causes pro-inflammatory gene activation when bound to ligands	(278–280)
Proteomics			
Clusterin, Opticin, Prostaglandin-H2 d-isomerase, complement C3, IGLL5, Vitronectin	nAMD; RRDCD; retinal vein occlusion	Clusterin: Protein chaperone that promotes cell survival and protection from apoptosis Opticin: Protein that binds to collagen fibrils and regulates fibril morphology, spacing, and organization plays a large role in the extracellular matrix Prostaglandin-H2 d-isomerase: regulates the constriction and dilation of blood vessels; stimulates platelet aggregation Complement C3: Plays a central role in activation of the complement system and involved in the innate immune system IGLL5: Involved in immune processes associated with cell death Vitronectin: Cell adhesion and spreading promoter; has membrane protecting effects	(259,276,282–285)
Intravitreal RNAs			
miRNAs, circRNAs	DR, AMD	circRNAs: May play a role in post-transcriptional regulation; assist in splicing of mRNA miRNAs: Assists in RNA silencing and post-transcriptional regulation of gene expression	(288,289)

^a^function has been summarized based on information in references, and from website search: uniprot.org and google.com

### Collection of Vitreous and Analytical Methodology

Vitreous samples are generally collected via vitreous taps from patients undergoing vitrectomy. Outpatient, needle aspiration procedures have also been used, but less commonly (247,248). Given the invasiveness of vitreous sampling, novel sampling approaches have recently been explored through collection of vitreous reflux after intravitreal injections (249,250). Cacciamani and colleagues performed vitreal reflux collection with different sampling techniques including Schirmer strips, microsponges, and millipore filters in nAMD patients with vitrectomy controls (249). Srividya and colleagues utilized Schirmer tear strips to collect the vitreous reflux of DME and PDR patients and compared total protein concentration to undiluted vitrectomy samples. Results showed similar total protein concentrations between the vitreous reflux and vitrectomy samples (P <0.05) with no tear contamination (250). Comparatively, micropipette and millipore sampling resulted in higher protein concentrations although the protein profiles were similar. Contrary to the previous study, Schirmer strips resulted in very low protein concentration. The authors selected the micropipette sampling as the most effective method based on protein concentrations and lack of contaminants.

The most common method of analysis for biomarkers in vitreous samples is enzyme-linked immunosorbent assays (ELISAs) and for some specific biomarkers there are commercially available assay kits. Multiplex bead array assays such as BD™ Cytometric Bead Array and Luminex xMAP ® technology are now commonly being utilized to maximize the utility of the vitreous sample and can measure multiple analytes simultaneously. In addition, fluorescence-based DIGE combined with MALDI-TOF MS has enabled accurate quantitation of multiple proteins (251). For metabolomic analysis, high-resolution ^1^H-nuclear magnetic resonance (NMR) spectroscopy has been used to determine metabolic profiles in vitreous for defining disease states (252). Proteomic and genomic analysis techniques have been discussed in previous sections and are also used to analyze biomarkers in vitreous samples.

### Biomarkers in Vitreous

#### Angiogenic and Anti-angiogenic Markers: VEGF, PIGF, PDGF, PEDF

Vascular endothelial growth factor (VEGF), is an angiogenic and vasopermeable factor that has been identified as an important pathophysiologic mediator in the development and maintenance of intraocular neovascularization seen in neovascular eye disease (253,254). The success of anti-VEGF as a therapeutic target (Lucentis, Eylea) has prompted many investigators to explore other potential molecular biomarkers found in the vitreous that are thought to contribute to ocular disease states, with many studies measuring VEGF levels in tandem in attempt to draw pathophysiologic correlations as discussed in the subsequent sections. In addition to becoming the focus of neovascular eye disease treatment paradigm, investigators have studied VEGF levels as indicators of disease prognosis and severity, especially in diabetic retinopathy (DR).

In PDR patients, numerous studies have investigated the predictability of intravitreal VEGF levels as indicators of disease prognosis after vitrectomy. In one study, the intravitreal VEGF levels in eyes from 50 PDR patients that underwent vitrectomy were compared to normal control (255). Results showed that intravitreal VEGF levels in the eyes with progression (n=10) of PDR were significantly higher than stabilization (n=10) or regression (n=30) phase of the disease. This correlation between intravitreal VEGF and severity of PDR was also investigated in another study with similar average intravitreal VEGF levels in PDR patients and controls (256). The investigators concluded that intravitreal levels of VEGF may be a risk factor for progression of PDR by determining that the odds of progression of PDR after vitrectomy were increased by 1.539 times for every 100 pg/ml increase of intravitreal VEGF concentration. Aside from PDR progression, role of intravitreal VEGF in visual acuity has also been investigated with mixed results. One study in 114 PDR patients segregated into high-VEGF (≥5000 pg/mL) and low-VEGF (<5,000 pg/mL) groups, were compared after vitrectomy (257). The postoperative logMAR visual acuity was significantly worse in the high-VEGF group than in the low-VEGF group but there was no significant difference in preoperative status between the groups. In addition, the frequency of postoperative complications that developed within 24 months after surgery was significantly greater in the high-VEGF group. These findings indicate the patients need to be carefully monitored during the postoperative course. These results differed from a previous study which did not find an association between levels of VEGF and visual acuity (258). While evaluating results from these studies, it is important to take into consideration the different VEGF collection and analytical methods, inclusion/exclusion criteria, and statistical methodology. Another study evaluated VEGF profile in a spectrum of ischemic retinopathies, including neovascular glaucoma. Kovacs *et al*. characterized VEGF and other angiogenic factors in non-diabetic (non-DM), diabetic (DM), PDR, and neovascular glaucoma (NVG) (259). Results showed that significant changes in VEGF levels were observed between the DM group and PDR group (p=0.013) and changes were not significant between the PDR and NVG group. Interestingly, placental growth factor (PIGF) was also studied and was the only protein that showed statistically significant increases with increasing levels of retinal ischemia (p=0.006 between PDR and NVG group). A recent study by Al Kahtani *et al*. also investigated PIGF and its relation to PDR severity, VEGF levels, and bevacizumab treatment (260). In this study, PIGF levels correlated to VEGF levels in active PDR and the PIGF levels were significantly greater in active PDR group *versus* inactive PDR group, suggesting that PIGF plays a critical role in PDR pathogenesis. Interestingly, vitreous levels of PlGF were not affected by preoperative treatment with bevacizumab.

Concentration of VEGF receptors in vitreous has also been studied in the form of soluble VEGF receptor (sVEGFR) in both PDR and AMD patients. Huber and Wachtlin demonstrated increased sVEGFR levels in both PDR and AMD with choroidal neovascularization when compared to control (261). This increase was accompanied by decreases in pigment epithelium-derived factor (PEDF) and increases in angiopoietin 2, which revealed the pro-angiogenic potential. However, the authors postulate that increased sVEGFR levels observed were a result of anti-angiogenic system being activated concomitantly. Noma and colleagues demonstrated a correlation between intravitreal VEGF and sVEGFR-1 and sVEGFR-2 in PDR patients with and without neovascular glaucoma (262).

Platelet-derived growth factors (PDGFs) are potent mitogens that are released by platelets. They are thought to contribute to ocular neovascularization by initiating the pericyte coating process on capillaries which leads to stabilization and endothelial cell survival (263). Three isoforms of PDGF (-AA, -AB, and -BB) were investigated in the serum and vitreous of patients with non-proliferative diabetic retinopathy (NPDR; n=15), PDR patients (n=31), and non-diabetic patients (n=15) (264). PDGF-AA and -AB levels were significantly increased in the vitreous of patients with NPDR compared to control, but levels of PDGF-BB were decreased. In PDR patients, all isoforms of PDGF were increased compared to control and NPDR. Anti-PDGF therapies have been assessed clinically in AMD patients as combination therapies with anti-VEGFs. Two recent examples include Regeneron’s Phase 2 CAPELLA study which assessed rinucumab/aflibercept *versus* aflibercept monotherapy and Ophthotech’s two pivotal phase 3 studies (OPH1002 and OPH1003) comparing Fovista/ranibizumab *versus* ranibizumab monotherapy. Both Fovista and rinucumab combination therapies failed to demonstrate visual outcome improvement over anti-VEGF monotherapy (265). Although PDGFs were unable to be established as a therapeutic target, they clearly play a role in understanding disease pathophysiology in DR.

PEDF is an endogenous antiangiogenic factor found in human RPE cells (266). As previously mentioned, decreased levels of this marker in vitreous have been observed in AMD patients (261). Conversely, significant increases of PEDF levels (1.45 times) in PDR patients were observed by Chernykh *et al*. compared to nondiabetic controls (267). VEGF was also evaluated and was 17-times higher in PDR patients *versus* control. The authors propose that the increases in opposing angiogenic and antiangiogenic factors may suggest disturbances of compensatory mechanism in angiogenesis regulation in PDR.

#### Inflammatory Cytokines and Neurotrophins

Inflammatory mediators have been studied in a variety of ocular disorders postulated to have an inflammatory component. There is an array of literature supporting increased levels of various inflammatory mediators (e.g. IL-6, IL-8, TNF- α) in DR and has been systematically reviewed (268). One study investigated the association of both inflammatory cytokines and neurotrophin biomarkers in various stages of DR (269). The inflammatory proteins of focus were IL-1β, IL-6, IL-8, and TNF-α, although others were studied. Both PDR and NPDR patients had higher levels of the above indicated inflammatory proteins, suggesting that these inflammatory markers are elevated in early stages of DR and could serve as useful markers of disease state. The neurotrophins of focus were, neurotrophin-3 (NT-3), neurotrophin-4 (NT-4), nerve growth factor (NGF), brain derived neurotrophic factor (BDNF), ciliary neurotrophic factor (CNTF), and glial cell line-derived neurotrophic factor (GDNF). Similar to the inflammatory mediators, the neurotrophin levels were higher in both PDR and NPDR *versus* nondiabetic eyes, with NPDR having higher concentrations than those in PDR. The neurotrophins and cytokine levels correlated in both the NPDR (P=0.02) and PDR (P=0.001) groups. The authors suggest that the increased inflammatory mediators naturally present in DR patients result in increased production of protective neurotrophins from retinal cells as a response. These findings signify the role of the inflammatory cytokines in early DR. Kovacs *et al*.. also investigated interleukins (IL-6 and IL-8) in non-DM, DM, PDR, and NVG patients, and identified IL-6 and IL-8 as potent drivers for NVG (259). In addition, the authors demonstrated that levels of IL-8 significantly increased between DM and PDR, and PDR and NVG groups. Another study attempted to correlate intravitreal IL-6 levels with thrombin-antithrombin III (TAT) complex in the vitreous of patients with different vitreoretinal pathologies: macular hole (MH)/epiretinal membrane (ERM; n = 26); rhegmatogenous retinal detachment (RRD; n = 32); and PDR (n = 20) (270). A significant difference was found in the vitreal IL-6 and TAT levels between the MH/ERM group and both the PDR and RRD groups (P < 0.001 for all). Different relationships between the IL-6 and TAT levels were revealed in patients with different ocular pathologies. These differences could not be attributed to the presence of diabetes as well as to variation in patients’ age or sex and therefore additional studies are needed to understand the dependence of these 2 biomarkers in pathophysiology of different retinal diseases

#### Hemodynamic Markers: Nitric Oxide and Endothetlin-1

Hemodynamic changes that result in increased blood flow to the retina have been correlated to retinopathy and are implicated in the pathogenesis of various vitreoretinal diseases. The main hemodynamic markers that have been studied are NO and endothetlin-1 (ET-1), with both factors known to be activated by elevated glucose levels (271,272). NO functions as a vasodilator and its expression can lead to increased cell death and impaired proliferation (271). ET-1 is a vasoconstrictor and is thought to have profibrotic and proliferative effects on vasculature (273). The vitreous levels of both hemodynamic factors were assessed in a study of 15 NPDR and 5 PDR patients compared to 5 control patients with a full thickness macular hole (274). The investigators did not find any differences in NO levels between groups but found increases in ET-1 levels in the PDR group. The results should be interpreted with caution due to the small number of patients studied.

#### Acute Phase Factors

Acute phase factors such as α2-macroglobulin, C-reactive protein (CRP), haptoglobin, ferritin, fibrinogen, serum amyloid P, procalcitonin, tissue plasminogen activator, and pentraxin 3 are up-regulated or down-regulated in inflammation (275). In one study, Kimura *et al*.. demonstrated that serum amyloid P, procalcitonin, ferritin, and fibrinogen were increased in the vitreal fluid of DME patients *versus* control subjects with intact epiretinal membrane (275). Among the increased acute phase factors in DME patients, correlations with visual acuity, central retinal thickness, and intravitreal VEGF levels were examined. Fibrinogen and procalcitonin were inversely correlated to visual acuity before and after surgery but were not correlated to central retinal thickness or intravitreal VEGF levels. The investigators suggest that given these results, acute phase factors may contribute to both the pathogenesis and prognosis of DME. Although proteomics is discussed in a later section, it is important to note here that vitreal fibronectin and fibrinogen expression was recently studied in PDR patients with and without intravitreal injection of anti-VEGF prior to vitrectomy (276). Quantitative proteomics analysis showed that the intravitreal injection group had higher signal intensities for fibronectin, and fibrinogen α, β and γ chains, when compared to the non-intravitreal injection group validated by ELISA. The authors suggested that increases in these acute phase factors result in fibrin-fibronectin complexations which contribute to the development of tractional retinal detachment in patients receiving intravitreal injections of anti-VEGF. In addition to fibrinogen and procalcitonin, Pentraxin-3 has also been studied and shown to be increased in vitreal concentration of PDR patients (277). Acute phase factors offer a differentiated target to the standard angiogenic approach in understanding the pathogenesis and prognosis of DR.

#### Advanced Glycation End Products

Advanced glycation products (AGEs) are induced by high glucose levels and have been linked to higher vitreous levels in diabetic patients *versus* non-diabetics and implicated in the pathogenesis of DR (278). Recently, Katagari *et al*.. investigated intravitreal glyceraldehyde-derived advanced glycation products (glycer-AGEs), also known as toxic-AGEs (TAGEs) in NPDR, PDR with simple vitreous hemorrhage (VH), and PDR with fibrovascular proliferative membrane (FVM) patients with the aim of correlating levels to DR disease severity and other angiogenic factors (i.e VEGF, IL-8, leptin, PIGF, endoglin, and fibroblast growth factor (FGF)-2) (279). Both Glycer-AGE and VEGF levels were higher in the FVM group *versus* the VH and NPDR groups. Although there was no correlation between glycer-AGE and VEGF levels, this study provides insight into the correlation of glycer-AGE pathway and DR disease severity and the authors suggest this marker as another potential therapeutic target to accompany anti-VEGFs. The soluble receptor for advance glycation end products (sRAGE) has also been studied in PDR by Katagiri *et al*.. with the aim of correlating its vitreous levels with VEGF levels, renal function, and PDR prognosis (280). They demonstrated significant correlations between sRAGE and renal function in DR progression, an important finding due to high prevalence of chronic kidney disease (CKD) among diabetics (281).

#### Proteomics

Proteomics have been used to identify protein signatures in a variety of ocular disease states such as DR, AMD, RRD, and retinal vein occlusion (RVO) that can serve as potential biomarkers. Li and colleagues conducted a proteomic analysis with vitreous samples collected from PDR and idiopathic macular hole (IMH) patients to further understand the molecular patho-mechanisms of PDR (282). By performing mass spectrometry-based label-free quantitative proteomics, they were able to identify 610 intravitreal proteins in total with 64 identified as unique to PDR and 212 to IMH patients. The investigators also identified 52 proteins that were up-regulated and 10 that were down-regulated in PDR *versus* IMH patients. Additionally, the authors identified 8 proteins most significant in PDR patients and suggested through protein function analysis that immunity and transport related proteins might be associated with PDR. Nobl *et al*.. conducted a proteomic analysis with vitreous samples from treatment naïve neovascular AMD (nAMD) patients with varying degrees of clinical presentation (283). By utilizing electrophoresis coupled to mass spectrometry (CE-MS) as well as LC-MS/MS, a total of 101 different proteins were identified. Among the proteins, clusterin, opticin, PEDF, and prostaglandin-H2 d-isomerase were identified and validated through receiver operating characteristic (ROC) and ELISA measurements as significant in nAMD. In another study, Wu *et al*.. investigated intravitreal fluid of RRD associated with choroidal detachment (RRDCD) by utilizing iTRAQ combined with nano-LC-ESI-MS/MS along with bioinformatic analysis (284). They identified 103 different proteins, with 54 that were up-regulated and 49 that were down-regulated in RRDCD. They utilized Kyoto encyclopedia of genes and genomes (KEGG) pathway analysis to classify the complement and coagulation pathways that were most common among the proteins found. Reich *et al*.. investigated vitreous samples of previously untreated patients with RVO by utilizing CE-MS and proteomic analysis (285). They identified 94 proteins in total with five as significant markers for RVO including complement C3, clusterin, opticin, Ig lambda-like polypeptide 5 (IGLL5), and vitronectin. These proteins that were identified as significant, were validated through ROC and ELISA measurements.

#### Metabolomics

As the newest “omic”, metabolomics is the study of metabolites that are endogenous and/or exogenous within the biological systems and has recently been utilized more frequently in studying cellular metabolism changes that occur during ocular disease pathogenesis (286). Although the plasma is more commonly studied, metabolomics approaches of vitreous humor have been applied in vitreoretinal disorders. For example, Haines *et al*.. investigated vitreous samples of PDR and RRD patients by utilizing untargeted mass-spectrometry-based metabolomics (287). They identified the changes in glucose metabolism and purine metabolism, and activation of the pentose phosphate pathway as significant in PDR but did not find any significant metabolic processes in RRD patients when compared to control. The changes observed in PDR patients point to oxidative stress as a key player in pathophysiology of PDR.

#### Intravitreal RNAs

Circular RNAs are a novel class of RNA transcripts, which are known to regulate gene expression. Intravitreal micro RNAs (miRNAs) and circular RNAs (circRNAs) expression profiling has been investigated in DR and AMD, respectively. Zhang *et al*.. investigated the expression and clinical significance of circRNAs in DR patients (288). Additionally, the interactions between circRNAs and miRNAs were investigated. Results showed upregulation of Circ_0005105 which was determined to be implicated in endothelial angiogenic function and inhibition of miR-519d-3p activity. Ménard *et al*. utilized non-biased microRNA assays and individual TaqMan qPCRs to profile intravitreal miRNAs in AMD patients and identified disease-associated increases and decreases in a set of miRNAs that were correlated to plasma levels (289). These studies show the emerging potential of miRNAs and circRNAs in understanding AMD and DR pathophysiology.

Considering the invasive nature of obtaining a vitreous sample, it is imperative that multiple biomarkers are assessed in a given sample. Advances in analytical technologies over the past decade with advent of proteomic and metabolomic analysis, has made it possible to maximize the utility of the vitreous sample and thus further our understanding of the posterior segment disorders based on relevant biomarkers.

## Ocular Biomarkers – Challenges and Opportunities: Authors’ Perspective

Biomarkers have been extensively used in clinical trials to assist in diagnosis of disease, stratification of patient population, understanding disease pathophysiology and as an exploratory measure of treatment response. In the ocular space, there are few biomarkers that have FDA approved tests for their measurement such as InflammaDry (measure levels of MMP-9 in tears) and Advance Tear Diagnostics (testing of tear levels of lactoferrin), which are primarily used for diagnosis of a disease. Most of the biomarkers are exploratory in nature but are still valuable tools used to answer several questions as we traverse the drug development pathway. The ocular matrices most commonly sampled in humans in clinical trials for evaluation of biomarkers are tears, conjunctiva, aqueous humor and vitreous. Of these, tears, AH and vitreous are fluid matrices, with the key challenge being collection of adequate sample volume. In addition, AH and vitreous sample collection during a study is very limited, due to the invasive collection procedure. Due to invasiveness of the technique there is additional risk to the patient in terms of damaging other ocular tissues. Hence once a sample is obtained, it is critical to have optimized processing and analytical methods to maximize the sample utility in order to evaluate a variety of biomarkers. With regards to tear collections, samples can be obtained more frequently, but it is important to avoid activating reflex tearing which can alter the composition of the sample, making the results difficult to interpret. In addition, factors such as use of artificial tears, contact lens, collections from closed *versus* open eyes and different collection techniques (eg. Schirmer strips, capillary) can also impact tear composition and should be considered. In conjunctiva, which is a solid tissue matrix, collection of adequate cells to determine biomarkers becomes challenging. Moreover, maintaining the integrity of these cells such that useful information can be gathered several hours to days post collection, serves as another hurdle. Systematic studies have been performed to evaluate sample integrity and reproducibility with regards to certain established biomarkers such as HLA-DR and goblet cell density. These studies have demonstrated that the sample storage duration is limited to a few days to a couple of weeks. Our internal data suggests that a maximum of 3 weeks storage time is suitable for goblet cell density evaluation in IC samples, with longer duration potentially compromising the cell integrity (unpublished data). Given these constraints, timely evaluation of the biomarkers in conjunctival cells is critical. Despite the challenges, during the past decade, the IC sampling has proved to be safe, noninvasive, highly efficient and reproducible technique which has significantly advanced biomarker evaluation in conjunctiva.

A common theme that has evolved in light of these challenges is the need to develop guidelines for standardization of collection methods, processing and storage of these ocular samples such that the data collected is comparable across studies and increases confidence in the measured biomarkers. Nevertheless, the advances in analytical techniques has led to new opportunities to utilize a single sample for multiple biomarker assessments and result in data that is more accurate and robust. Improved sensitivities in protein-based and gene-based technologies have made it possible to evaluate multiple biomarkers in a small sample volume of tears, AH or vitreous and conduct transcriptome-wide gene expression analysis in conjunctiva. Relatively novel technology such as IVMC has made it possible to assess cellular changes in cornea and conjunctiva, without any sample collection. In addition, despite the current challenges around cost, standardization of technique, IVMC looks promising for biomarker evaluation in future. Biomarker evaluation in ocular matrices has significantly advanced our knowledge of the ocular disease pathophysiology, unlike any evaluation would have in surrogate matrices such as blood or urine. It has provided tools to assess disease severity, explore treatment effect and determine correlations with clinical endpoints. In several cases, biomarker evaluation has helped in identifying potential targets as treatment options for future development. Biomarkers continue to be useful measures of determining target engagement in early stages of drug development and advancement in technologies will lead to more validated biomarkers that can serve as surrogate endpoints in clinical trials. Moreover, ongoing progress in the field of ocular biomarkers will enable us to incorporate a personalized approach in treatment paradigms which has the potential to significantly benefit patients.

## ABBREVIATIONS

AGP: A-1-acid glycoprotein1
AH: Aqueous humor
AKC: Atopic keratoconjunctivitis
AMD: Age-related macular degeneration
ANP: Atrial natriuretic peptide
ANXA1: Annexin 1
ANXA11: Annexin 11
APO AI: Apolipoprotein A1
APO CIII: Apolipoprotein C-3
APO E: Apolipoprotein E
ATD: Aqueous tear deficiency
ATX: Autotaxin
BDNF: Brain derived neurotrophic factor
BID: Twice daily
BNP: Brain natriuretic peptide
CALT: Conjunctival associated lymphoid tissue
CCL2/MIP-1: Chemokine (C-C motif) ligand 2/Monocyte chemoattractant protein 1
CCL3/MIP-1α: Chemokine (C-C motif) ligand 3/Macrophage inflammatory protein 1 alpha
CCL4/MIP-1β: Chemokine (C-C motif) ligand 4/ Macrophage inflammatory protein 1 beta
CCL5/RANTES: Chemokine (C-C motif) ligand 5 / Regulated on Activation, Normal T cell Expressed and Secreted
CCL11: Chemokine (C-C motif) ligand 11
CCL24: Chemokine (C-C motif) ligand 24
CCL26: Chemokine (C-C motif) ligand 26
CCR2: Chemokine (C-C motif) receptor 2
CCR5: Chemokine (C-C motif) receptor 5
CD3+: Cluster of differentiation 3 positive
CD4+: Cluster of differentiation 4 positive
CD8+: Cluster of differentiation 8 positive
CD44S: Soluble form of CD44 (Cluster of differentiation 44)
cGMP: Cyclic guanosine monophosphate
CGRP: Calcitonin-gene-related peptide
circRNA: Circular ribonucleic acid
CNTF: Ciliary neurotrophic factor
CsA CE: Cyclosporine cationic emulsion
CX3CL1: C-X3-C motif chemokine ligand 1 / Fractalkine
CXCL8: Chemokine (C-X-C motif) ligand 8
CXCL9: Chemokine (C-X-C motif) ligand 9
CXCL10: Chemokine (C-X-C motif) ligand 10
CXCL11: Chemokine (C-X-C motif) ligand 11
CXCL12: Chemokine (C-X-C motif) ligand 12
CXCR4: Chemokine (C-X-C motif) receptor 4
DED: Dry eye disease
DM: Diabetic
DME: Diabetic macular edema
DR: Diabetic retinopathy
ECP: Eosinophilic cationic protein
EGF: Epidermal growth factor
EGFR: Epidermal growth factor receptor
ELAM-1: Endothelial leukocyte adhesion molecule-1
ES: Exfoliation syndrome
ET-1: Endothelin-1
GDNF: Glial cell line-derived neurotrophic factor
glycer-AGE: Glyceraldehyde-derived advanced glycation products
GM-CSF: Granulocyte macrophage-colony stimulating factor
GPC: Giant papillary conjunctivitis
GPX: Glutathione peroxidase
GVHD: Graft versus host disease
HEL: Hexanoyl-lysine
HGF: Hepatocyte growth factor
HLA-DR: Human leukocyte antigen-D-related
4-HNE: 4-hydroxy-2-nonenal
IC: Impression cytology
ICAM-1: Intercellular adhesion molecule 1
IFN-γ: Interferon-gamma
IgE: Immunoglobulin E
IGLL5: Immunoglobulin G lambda-like polypeptide 5
IL-1: Interleukin-1
IL-1α: Interleukin-1 alpha
IL-1β: Interleukin-1 beta
IL-3: Interleukin-3
IL-4: Interleukin-4
IL-6: Interleukin-6
IL-8: Interleukin-8
IL-10: Interleukin-10
IL-17: Interleukin-17
IL-17A: Interleukin-17A
IL-17F: Interleukin-17F
IL-22: Interleukin-22
IOP: Intraocular pressure
IPA: Ingenuity pathway analysis
ITN: Intranasal tear neurostimulator
IVMC: In vivo confocal microscopy
LCN-1: Lipocalin-1
LFU: Lacrimal functional unit
LOXL1: Lysyl oxidase-like 1
LPA: Lysophosphatidic acid
LPRR3: Lysozyme proline-rich protein 3
LPRR4: Lysozyme proline-rich protein 4
MDA: Malondialdehyde
MGD: Meibomian gland dysfunction
miRNAs: Micro RNAs
MMP-9: Matrix metalloproteinase-9
MUC5AC: Mucin 5 subtype AC, oligomeric mucus/gel forming
MUC16: Mucin 16
nAMD: Neovascular age-related macular degeneration
NAMPT: Nicotinamide phosphoribosyltransferase
NGF: Nerve growth factor
NGS: Next-generation sequencing
NK: Natural Killer
NMDA: N-methyl-D-aspartate receptor
NO: Nitric oxide
NPDR: Non-proliferative diabetic retinopathy
NSS-KCS: Non- Sjögren syndrome-associated keratoconjunctivitis sicca
NT-3: Neurotrophin 3
NT-4: Neurotrophin 4
NVG: Neovascular glaucoma
OCT: Optical coherence tomography
OGVHD: Ocular graft versus host disease
ONH: Optic nerve head
PACG: Primary angle-closure glaucoma
PAS: Periodic acid schiff reagent
PAX6: Paired-box protein 6
PDGF: Platelet-derived growth factor
PDR: Proliferative diabetic retinopathy
PEDF: Pigment epithelium-derived factor
PEX: Pseudo-exfoliation glaucoma
PIGF: Placental growth factor
PIP: Prolactin inducible protein
POAG: Primary open angle glaucoma
PRP4: Pre-mRNA Processing Factor 4
QD: Daily
RGCs: Retinal ganglion cells
RNFLT: Retinal nerve fiber layer thickness
RRDCD: Rhegmatogenous retinal detachment associated with choroidal detachment
RRD: Rhegmatogenous retinal detachment
RVO: Retinal vein occlusion
S100 proteins: Calcium activated signaling proteins
SC: Schlemm’s canal
SM: Squamous metaplasia
sNCAM: Soluble neural cell adhesion molecule
SOD: Superoxide dismutase
SPRR1B: Small proline rich protein 1B
sRAGE: Soluble receptor for advance glycation end products
SS: Sjögren’s Syndrome
sVCAM-1: Soluble vascular cell adhesion molecule-1
sVEGFR: Soluble vascular endothelial growth factor receptor
TAGEs: Toxic advanced glycation products
TAO: Thyroid-associated ophthalmopathy
TAT: Thrombin-antithrombin III
TBUT: Tear film break-up time
T_CM_: T lymphocytes central memory
T_EM_: T lymphocytes effector memory
TFBUT: Tear film breakup time
TGF-β: Transforming growth factor β
TGF-β1: Transforming growth factor β1
TGF-β2: Transforming growth factor β2
Th1: T helper 1 cell
Th17: T helper 17 cell
TM: Trabecular meshwork
TNF-α: Tumor Necrosis Factor- α
TO: Thyroid orbitopathy
TTR: Transthyretin
VCAM-1: Vascular cell adhesion protein 1
VEGF: Vascular endothelial growth factor
VIP: Vasoactive intestinal peptide
VKC: Vernal atopic conjunctivitis
